# A Dynamic Factor and Neural Networks Analysis of the Co-movement of Public Revenues in the EMU

**DOI:** 10.1007/s40797-021-00155-2

**Published:** 2021-05-24

**Authors:** Cosimo Magazzino, Marco Mele

**Affiliations:** 1 Department of Political Sciences, Roma Tre University, Rome, Italy; 2 Roma Tre University, Rome, Italy

**Keywords:** Fiscal sustainability, Public revenues, European Monetary Union, Dynamic factor, Neural networks, C22, C23, E62, H20

## Abstract

This paper shows that the co-movement of public revenues in the European Monetary Union (EMU) is driven by an unobserved common factor. Our empirical analysis uses yearly data covering the period 1970–2014 for 12 selected EMU member countries. We have found that this common component has a significant impact on public revenues in the majority of the countries. We highlight this common pattern in a dynamic factor model (DFM). Since this factor is unobservable, it is difficult to agree on what it represents. We argue that the latent factor that emerges from the two different empirical approaches used might have a composite nature, being the result of both the more general convergence of the economic cycles of the countries in the area and the increasingly better tuned tax structure. However, the original aspect of our paper is the use of a back-propagation neural networks (BPNN)-DF model to test the results of the time-series. At the level of computer programming, the results obtained represent the first empirical demonstration of the latent factor’s presence.

## Introduction

The size of government should be measured by the resources it commands. Among alternative measure of government size, we can find the public revenue/gross domestic product (GDP) ratio. For decades there has been an intense debate regarding the relationship between government size and economic growth. Surprisingly, in the last 50 years the dynamics of government size in the European Monetary Union (EMU) countries follows a common trend.

Tax distortions are relevant for economic growth in the long-run. Recent empirical studies underline a clear link between tax structure and economic growth. However, this link is questioned by Xing (2012), who demonstrated that the evidence for significant tax structure effects depends on long-run parameter homogeneity restrictions.

One of the primary objectives for the creation of the EMU was to economically spur the growth process by creating cohesive policies to steer the movement of public revenues. The co-movement of public revenues in the EMU depends on the fiscal policies established by the member countries. According to Hagen and Waller (2013), EMU has come across numerous challenges, in the long-run, trying to harmonize the economic welfare across the borders of the members. Economic factors such as tax harmonization, public revenue equalization, and also the fiscal authority policies hamper the different measures and procedures implemented (Barrell et al. 2008). While the significant countries and key stakeholders want to maintain the status quo, the results prove a challenging position for the policymakers.

The aim of this paper is to investigate the (eventual) common factor that has driven the co-movement of public revenues in the EMU from 1970 to 2014. To the best of our knowledge, this is the first study that analyzes public revenues in a monetary union highlighting an unobserved factor. Moreover, the novelty of this research is to complement the time-series analysis with a machine learning (ML) approach, as a robustness check. The discovery of a latent factor driving the public revenues of the EMU member countries would be interesting because it would have far-reaching political implications. If the national economies of this currency area are also interconnected in terms of revenues, then smaller efforts would be needed to complete the monetary union, and harmonizing public debt/GDP ratios remains the only real obstacle to hinder centralizing fiscal policies (De Grauwe 2018). In fact, finding some common factor in the government revenues of the member countries of a currency area can bring this area closer to satisfy the conditions for becoming an Optimal Currency Area (OCA) (Mundell 1961; McKinnon 1963; Kenen 1969; De Grauwe 2018; Baldwin and Wyplosz 2019). This should be particularly relevant in the light of the recent crises (subprime mortgages; sovereign debt; Coronavirus Disease).

As regards the empirical strategy, we first apply a dynamic factor model (DFM) to estimate an unobserved common component in the government revenues/GDP ratios for EMU-12. Furthermore, we attempt to provide an explanation of the unobserved factor. Then, we test the results obtained through a time-series model by a procedure of artificial neural networks (ANNs) in a DFM. To the best of our knowledge, this is the first paper that applies this procedure in a socio-economic framework. In fact, it is used by meteorological sciences to predict the dynamics of climate change. Therefore, we used the same procedure to study in a neural network (NN) the dynamic effects of the economic cycle on the revenues of the countries taken into consideration. Moreover, the ML model, following Hofmann (2001) mathematical settings, generated a latent factor in a NN for the first time. It represents the result of multiple algorithms transcribed in Java and Oryx that have interpreted the nexus of matrices in a Markov process as a single composite NN algorithm. The results obtained, after carrying out several different tests, confirm the time-series ones.

The novelty of our latent factor estimation model can be split into two elements. The first is attributable to the analysis model. In fact, the very few previous studies (Pan and Wang 2012; Calzolari and Halbleib 2018; Chen et al. 2020) use an inferential model to estimate the latent factor. There is always an assumption based on these analyzes. It identifies a set (*K*) of common factors to extrapolate, through principal component analysis-probabilistic latent semantic analysis (PCA-PLSA), one or more latent factors. The author then interprets the process using the logical assumption of inference concerning mere empirical conjecture. This last statement fits precisely with the second element that distinguishes our estimate from that usually used in literature: the software used.

The study proceeds as follows. Besides this introduction, in Sect. 2 we show a state-of-the-art, focusing on some relevant empirical results provided in the economic literature. Section 3 describes the applied methodology and data, while in Sect. 4 the results are presented and discussed. Section 5 provides further explanations on the latent factor analyses. Finally, Sect. 6 gives conclusions, policy implications, and suggestions for future research.

## Empirical Literature

In determining the co-movement of public revenues in the EMU, Frieden and Eichengreen (1998) argued that rigid prices, wages, and the ever constant low labor mobility hinders the co-movement of public revenues within the EMU as a result of asymmetric shocks that cause an immediate difference in monetary flow and business cycles. In their findings, the researchers found a direct relationship between public revenues movements and dynamic asymmetric shocks. The risk factors involved scares away businesses, thus reducing revenue flows.

Since the co-movement of public revenues largely depends on the current economic structures amongst EMU members, the analytical factors depend on the mechanisms established. The co-movement of public revenues, according to Sims and Zha (2006), solely depends on the policies of each sovereign government. The results suggest that short-term effects of the revenue flows may facilitate in determining the long-term effects.

As pinpointed by Kopits and Symanski (1998), the policies guiding the EMU lack an official fiscal authority to coordinate them. The organization majorly relies on existing institutions such as the Economic and Financial Affairs Council (ECOFIN). The lack of a central regulatory institution means that the coordination process of the public revenues movement remains ineffective.

Katz et al. (1983), Koester and Kormendi (1989), and Agell et al. (1997) found that the average tax rate has no effect on economic growth. Other seminal papers by Landau (1983), Kormendi and Meguire (1985), Skinner (1988), Grier and Tullock (1989), and Engen and Skinner (1992) found a negative correlation between government budgetary policy and economic growth. Cass (1965) and Koopmans (1965) showed that fiscal policy changes affect the equilibrium level of GDP, with only transitional growth effects. Cameron (1978) found a growing tendency for increases in taxes and social spending to erode the rate of capital formation. Miller and Russek (1997) underlined that property, income and even sales taxes decrease income and employment growth. Yamarik (2000) showed that personal income tax rates decrease long-run per capita income growth. Ojede and Yamarik (2012) found that property taxes lower both short-run and long-run economic growth, sales taxes lower long-run growth, while income taxes have no short-run or long-run impact. Arnold et al. (2011) found a ranking in terms of the effects of different types of taxes on the level of income per capita in the long-run. Dalena and Magazzino (2012) showed that, for each time period, the policy adopted by Italian governments reflected the prevailing paradigm of public finance.

Zagler and Dürnecker (2003) provided a useful survey of the literature on fiscal policy and economic growth.

Recently, Brady and Magazzino (2018b) extended the research on the fiscal sustainability of the EMU countries in the 1980–2015 period. Their results indicate that the solvency condition would be satisfied for the EMU countries.

Analyzing the policies set indicates that revenue movement within EMU directly depends on the plan. According to Ostrup (2000), the sensitivity of the economy directly affects money flow. The systematic obligation of the economy is the monetary policy of each EMU member. The current framework does not provide EMU with total control or policymaking, thus hindering money flow. Concerning the analytical approach, there are always the dominant economies as a result of constrained policies. The effects of the determiners of the economies show that non-policy shocks have less impact on the entire process.

Another factor that determines the co-movement of public revenues is jurisdiction to employment. Fostering policies that restrict flexibility to the movement of people find its way in inhibiting inequality. Roel et al. (2001) concluded that optimal stabilization is a factor that EMU governments should consider. Analytical factors and demand shocks indicate that revenue flow requires incentives to achieve stability in the region. Such a scenario results in winners and losers in the long-run (Ostrup 2000). The economic cycle may also vary from one region to the other; thus, intervention from the government and policymakers could cost and also create avenues for clearing such asymmetric and adverse effects.

According to Klein and Mittnik (2013), stabilizing policies generate the essence of revenue flow across orders of the member states. When all the factors are considered, then public revenues flow is restrained by the policymaking process.

The analysis of public revenues is also relevant from the perspective of fiscal sustainability. Several studies inspected fiscal sustainability for European countries. Caporale (1995), Artis and Marcellino (1998), and Afonso (2005) found substantial evidence against the sustainability of fiscal policy. On the other hand, Afonso and Jalles (2014) and Brady and Magazzino (2018c) showed results in line with sustainability hypothesis. Finally, mixed results are due to Vanhorebeek and Van Rompuy (1995), Papadopoulos and Sidiropoulos (1999), Uctum and Wickens (2000), Bravo and Silvestre (2002), Kollias and Paleologou (2006), Afonso and Rault (2010), Ahmad and Fanelli (2014), Mercan (2014), and Brady and Magazzino (2017). Futher details are shown by Molnár (2012), Magazzino and Lepore (2015), Forte and Magazzino (2016), Brady and Magazzino (2018b), and Magazzino et al. (2020).

In addition, many empirical studies analyzed the degree of heterogeneity of EMU economies. The most lingering question underlying most issues is to know if member states are similar enough to share the same currency in the long-run. Buti and Sapir (1998) underlined the importance of reducing the degree of heterogeneity in national labour market institutions and outcomes, and—at the same time—to put in place risk-sharing mechanisms capable of absorbing asymmetric shocks. Frankel and Rose (1998) presented empirical evidence that countries with more bilateral trade will feature higher business cycle correlations. Marcellino et al. (2000) evaluated the homogeneity of the EMU countries on the basis of the degree of similarity of the driving forces of their economies. Using a DFM for a sample on the 1982–1997 period, the results highlighted that the driving forces appear to be common across countries.

Following the Great Recession, Europe is once again debating the use of fiscal instruments for macroeconomic stabilization, with the experience of a monetary union with common fiscal shock absorbers, such as in the U.S., as a reference. Research on fiscal stabilization and risk-sharing in the U.S. was originally a benchmark for EMU (Sachs and Sala-i-Martin 1992; Von Hagen 1992; Bayoumi and Masson 1995). Nikolov and Pasimeni (2019) showed that the design of the budget, in particular the balance of revenue and expenditure, can maximise its stabilisation effect.

## Methodology and Data

Geweke (1977), Coppi and Zannella (1978), Watson and Engle (1983), and Stock and Watson (1989,1991) applied DFM in macro-econometrics. A DFM focuses on the spectral density matrix of a set of time series, whereas a static factor model explains the variance–covariance matrix among observed cross-sectional variables. Moreover, DFM also lessens the need for strong assumptions related to structural models. According to Engle and Kozicki (1993) the latent factor is a “common feature”.

If *R*_*it*_ denotes a measure of growth of public revenues for country *i* at time *t*, we can estimate the following DFM:

1 $$ \begin{gathered} R_{it} = \alpha_{i} F_{t} + \beta_{i} X_{t} + \, u_{it} \hfill \\ F_{t} = \, \rho F_{t - 1} + \, \upsilon_{t} \hfill \\ u_{it} = \, w_{i} u_{it - 1} + \, \varepsilon_{it} . \hfill \\ \end{gathered} $$

Thus, we identify three different components of public revenue growth in country *i*. *R*_*it*_ is the revenues-to-GDP ratio for country *i* at time *t*. *F*_*t*_ represents the unobserved common factor, which is the same across countries measuring the common effect on revenues for the EMU member states; *α*_*i*_ is a time-invariant impact coefficient, known as factor loadings and able to seizing the sensitivity to the latent factor on each country. The second component *X*_*t*_ is a common term in the EMU business cycle; *β*_*i*_ is a time-invariant impact coefficient. The last component *u*_*it*_ is a time-varying term specific to country *i*.

We write the DFM as a state-space model as suggested by the conventional approach, and use the Kalman filter to estimate the model. In particular, the state space form consists of a state vector, a transition equation, and a measurement equation that relates the state vector to observed variables.

To test the time series results, we use a supervised learning using a NN approach. According to Hopfield and Tank (1985), we use both linear and non-linear models of recurrent neurons to generate asymptotically stable processes capable of solving even inverse linear matrix equations. This mathematical model is an algorithm able to analyze the signal that goes to the inputs to the latent factor and, finally, to the target.

We structure an unconstrained matrix equation for an optimization problem as follows:

2 $$ \mathop {\min }\limits_{W} E\left[ {R\left( W \right)} \right] = \mathop \sum \limits_{i = 1}^{p} \mathop \sum \limits_{j = 1}^{q} e_{i,j} \left[ {r_{i,j} \left( W \right)} \right]. $$

In (1), $W = w$ and it is a matrix $m \times n$ where there is a solution matrix $M$. After, $r_{ij}$ is an objective function on $r_{ij} \left( W \right)$. So, in recurrent NN we have:

3 $$ \frac{{dw_{i,j} \left( t \right)}}{dt} = - \gamma \mathop \sum \limits_{k = 1}^{p} \mathop \sum \limits_{l = 1}^{q} \frac{{\vartheta r_{kl} \left[ {W\left( t \right)} \right]}}{{\vartheta w_{ij} }}f_{kl} \left\{ {r_{kl} \left[ {W\left( t \right)} \right]} \right.. $$

In (2), $$\gamma > 0$$, $$W\left( t \right) = r_{i,j} \left( t \right) $$ and it is an activation state matrix of the recurrent NN,$$ F\left( R \right) = f_{ij} \left( {r_{ij} } \right)$$ is an activation function matrix. Now, we solve the following matrix with $$i = 1,2..,m $$ and $$j = 1,2..,n$$:

4 $$ \frac{{dw_{i,j} \left( t \right)}}{dt} = - \gamma \left\{ {\mathop \sum \limits_{k = 1}^{p} \mathop \sum \limits_{l = 1}^{q} \frac{{\vartheta r_{kl} \left[ {W\left( t \right)} \right]}}{{\vartheta w_{ij} }}u_{kl} \left( t \right)} \right\} . $$

In (3), $$u_{kl} \left( t \right) = f_{kl} \left\{ {r_{kl} \left[ {W\left( t \right)} \right]} \right.$$.

So, we have:

5 $$ E\left( R \right) = \left( {\begin{array}{*{20}c} {e_{11} \left( {r_{11} } \right)} & {\left( {\begin{array}{*{20}c} {e_{12} } & \cdots & {r_{12} } \\ \vdots & \ddots & \vdots \\ {e_{22} } & \cdots & {r_{22} } \\ \end{array} } \right)} & {e_{1q} \left( {r_{1q} } \right)} \\ {e_{21} \left( {r_{21} } \right)} & r & {e_{2q} \left( {r_{2q} } \right)} \\ {e_{p1} \left( {r_{p1} } \right)} & {\begin{array}{*{20}c} {e_{p1} } & \cdots & {r_{p1} } \\ \vdots & \ddots & \vdots \\ {e_{p2} } & \cdots & {r_{p2} } \\ \end{array} } & {e_{pq} \left( {r_{pq} } \right)} \\ \vdots & \ddots & \vdots \\ {e_{p2} (r_{p2} )} & e & r \\ \end{array} } \right). $$

6 $$ F\left( R \right) = \left( {\begin{array}{*{20}c} {f_{11} \left( {r_{11} } \right)} & {\left( {\begin{array}{*{20}c} {f_{12} } & \cdots & {r_{12} } \\ \vdots & \ddots & \vdots \\ {f_{22} } & \cdots & {r_{22} } \\ \end{array} } \right)} & {f_{1q} \left( {r_{1q} } \right)} \\ {f_{21} \left( {r_{21} } \right)} & r & {f_{2q} \left( {r_{2q} } \right)} \\ {f_{p1} \left( {r_{p1} } \right)} & {\begin{array}{*{20}c} {f_{p1} } & \cdots & {r_{p1} } \\ \vdots & \ddots & \vdots \\ {f_{p2} } & \cdots & {r_{p2} } \\ \end{array} } & {f_{pq} \left( {r_{pq} } \right)} \\ \vdots & \ddots & \vdots \\ {f_{p2} (r_{p2} )} & f & r \\ \end{array} } \right). $$

The matrices (4) and (5) are the starting architecture of the recurrent NN for solving the bidirectional type linear matrix equations. Now, to generate the signals within a NN where latent factors may be present, we theoretically solve the following:

$E\left[ {R\left( W \right)} \right] \to \infty \;{\text{and }}\;W \to \infty .$ Thus, we have:

7 $$ \frac{{dE\left[ {R\left( W \right)t} \right] = }}{dt}\mathop \sum \limits_{k = 1}^{p} \mathop \sum \limits_{l = 1}^{q} \frac{{de_{kl} \left[ {r_{kl} W\left( t \right)} \right]}}{dt}u_{kl} \left( t \right) = \mathop \sum \limits_{k = 1}^{p} \mathop \sum \limits_{l = 1}^{q} \mathop \sum \limits_{i = 1}^{m} \mathop \sum \limits_{j = 1}^{n} \frac{{\vartheta e_{kl} }}{{\vartheta r_{kl} }}\frac{{\vartheta r_{ij} \left( t \right)}}{dt}, $$

8 $$ = \mathop \sum \limits_{k = 1}^{p} \mathop \sum \limits_{l = 1}^{q} \mathop \sum \limits_{i = 1}^{m} \mathop \sum \limits_{j = 1}^{n} \frac{{de_{kl} \left( {r_{kl} \left[ W \right)t} \right)])}}{{dr_{kl} }}\frac{{\theta r_{kl} }}{{\theta w_{kl} }}\frac{{dw_{kl} \left( t \right)}}{dt} $$

9 $$ = \mathop \sum \limits_{k = 1}^{p} \mathop \sum \limits_{l = 1}^{q} \mathop \sum \limits_{i = 1}^{m} \mathop \sum \limits_{j = 1}^{n} f_{kl} [r_{kl} \left( {W\left( t \right)} \right)] \frac{{\theta r_{kl} }}{{\theta w_{kl} }}\frac{{dw_{kl} \left( t \right)}}{dt} $$

10 $$ = - \frac{1}{\gamma }\mathop \sum \limits_{i = 1}^{m} \mathop \sum \limits_{j = 1}^{n} [\frac{{dw_{ij} }}{dt}]^{2} \left\{ {\begin{array}{*{20}c} { < 0 if \exists_{i,j} } \\ { = 0 if \forall_{ij} } \\ \end{array} } \right.. $$

Now, we use a Lyapunov matrix equation on the NN:

11 $$ \frac{dW\left( t \right)}{{dt}} = - \gamma \left[ {AU\left( t \right) + U\left( t \right)A^{t} } \right]. $$

12 $$ U\left( t \right) = F\left[ {A^{t} W\left( t \right) + W\left( t \right)A + Q} \right]. $$

13 $$ \frac{dw\left( t \right)}{{dt}} = - \gamma \mathop \sum \limits_{k = 1}^{n} \left[ {a_{ik} u_{kj} \left( t \right) + a_{jk} u_{ik} \left( t \right)} \right]. $$

14 $$ u_{ij} \left( t \right) = f_{ij} = \left\{ {\mathop \sum \limits_{k = 1}^{n} \left[ {a_{ki} w_{kj} \left( t \right) + a_{kj} w_{ik} \left( t \right)} \right] + q_{ij} } \right\}. $$

Using (12) and (13) in terms of algorithm in a binary system, it is obtained:

15 $$ A = \begin{array}{*{20}c} 0 & 1 & 0 & 1 & 1 & 0 \\ 1 & 1 & 0 & 0 & 1 & 0 \\ 0 & 0 & 0 & 0 & 0 & 1 \\ 0 & 1 & 1 & 0 & 0 & 0 \\ 0 & 0 & 0 & 1 & 1 & 1 \\ \end{array} . $$

16 $$ Q = \begin{array}{*{20}c} 1 & 0 & 1 & 0 & 0 & 1 \\ 1 & 1 & 0 & 0 & 1 & 1 \\ 0 & 0 & 1 & 0 & 1 & 0 \\ 1 & 0 & 1 & 0 & 0 & 0 \\ 0 & 1 & 0 & 0 & 1 & 1 \\ \end{array} . $$

As explained in Camba-Méndez and Kapetanios (2004), these methods are applicable to large datasets. This study uses the Penn world table (PWT) database of the Groningen Growth and Development Centre (GGDC) for real GDP and economic business cycle data.^1^ For data on government revenues, the source is the Annual Macro-Economic Database (AMECO) of the European Commission’s Directorate General for Economic and Financial Affairs (DG ECFIN) AMECO.^2^ We collected data for 12 selected EMU countries over the 1970–2014 years. The choice of using the revenue-to-GDP ratio instead of levels or first differences is based on different aspects:

- both previous papers on DFM used variables as GDP ratios (Albanese and Modica 2010; Pan and Wang 2012);
- the Maastricht Treaty sets the parameters of public finance variables in terms of their GDP ratios;
- dividing by GDP makes the series more comparable in a panel of countries.

Therefore, we are actually interested in a common factor able to capture the features of this ratio instead of the revenues dynamic.

## Empirical Results

We assume that the innovations in the two processes are uncorrelated across countries and over time. The autoregressive parameters, the factor loadings and the business cycle coefficients are fixed. It should be noted that while *X*_*t*_ is measured, *F*_*t*_ and *u*_*it*_ are two unobservables.

Previous research shows that a common component exists in the business cycle (Norrbin and Schlagenhauf 1996; Crucini et al. 2011). In order to partial out this common cyclical effect, we construct the measure of EMU business cycle following Crucini (1997), Albanese and Modica (2010), and Pan and Wang (2012): a weighted average of annual output growth rates of the EMU-12, where the weights are proportional to GDP (in PPP terms). Figure 13 in the Appendix plots this measure against time.

**Fig. 1:**
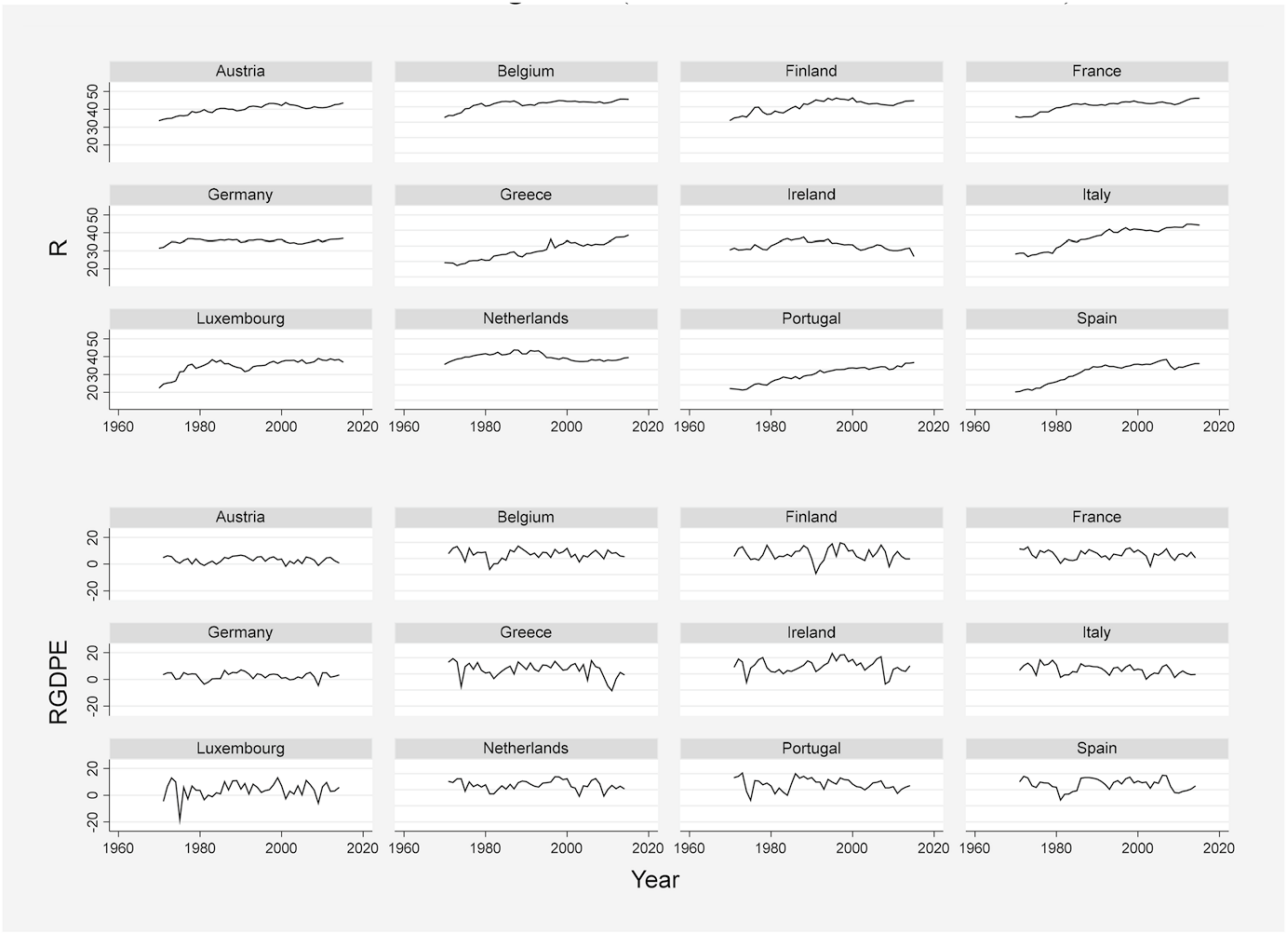
Total tax revenue and real GDP growth (12 EMU countries, 1970–2014). Sources: our elaborations on AMECO and PWT data

We estimate the DFM using the government revenues-to-GDP ratios for the EMU-12. Figure 1 reveals a common pattern. Since the series exhibits unit roots, we derive the first differences, and then standardize the series.

We include the EMU business cycle as a control variable in the DFM, and show the parameter estimates in Table 1. The coefficients of the unobserved factor are statistically significant at the 1% level for four countries (Finland, France, Luxembourg, and Portugal) and at the 10% level for five countries (Austria, Germany, Ireland, Netherlands, and Spain), while they are not statistically different from zero for Belgium, Greece, and Italy. It is worthy of attention that these three are also the countries in the EMU with the highest government debt-to-GDP ratio, and thus they have to face the most difficult challenges in order to restore their public finance equilibrium. Moreover, this finding can be attributed to the fact that these are the countries (together with Portugal and Spain) where the government revenues/GDP ratio increased the most during the sample period. Moreover, the unobserved factor is quite persistent, with a first-order autocorrelation of 0.8931 (at the 1% significance level). We also note that the EMU business cycle has a significantly positive impact on the revenues ratios for nine economies (except for Germany, Ireland, and Netherlands), reflecting a cyclical reaction to the economic cycle. Finally, using variance decomposition, we measure the relative contribution of the unobserved factor and EMU business cycle to the variation of government revenues ratios.

**Table 1:** Estimation results (12 EMU countries, 1970–2014)

Country	Impact coefficients		AR coefficient	% of variance of revenue ratio explained by common factors
	α	β	ρ	
Austria	− 0.3749* (0.1989)	0.8198*** (0.2719)		39.85
Belgium	− 0.1818 (0.1704)	0.7356** (0.3346)		55.58
Finland	− 0.5074*** (0.1384)	0.7026*** (0.2276)		32.22
France	− 0.5095*** (0.1579)	0.7693*** (0.2206)		47.97
Germany	− 0.3638* (0.2133)	0.6129 (0.4858)		45.91
Greece	− 0.4306 (0.2675)	0.7893** (0.3647)		29.12
Ireland	− 0.5050* (0.3388)	0.4054 (0.3287)		36.95
Italy	− 0.4280 (0.2844)	0.7110*** (0.2548)		49.11
Luxembourg	− 0.5088*** (0.1406)	0.8701*** (0.2319)		33.03
Netherlands	− 0.5279* (0.2677)	0.3786 (0.2532)		32.65
Portugal	− 0.4867*** (0.1680)	0.8680*** (0.2562)		28.02
Spain	− 0.6435* (0.3149)	0.9028*** (0.1627)		50.05
Latent factor			0.8931*** (0.1049)	

Robust standard errors in parentheses. ***p < 0.01, **p < 0.05, *p < 0.10

The unobserved factor could be represented by national efforts to exhibit public accounts in equilibrium, obeying to the Maastricht Treaty prescriptions on public deficit/GDP and public debt/GDP thresholds.

Now, we want to verify the results obtained with the time-series models previously used through a sophisticated model of NNs in ML. We use it in a separate but joint way, an algorithm transcribed in Java with Weka library, and subsequently reworked in the Oryx 2.0.8 software.

Theoretically, an NN is an adaptive system able to modify its structure (nodes and interconnections) based on both external data and internal information that connect and pass through the NNs during the learning and reasoning phase. In general, a biological NN receives external data and signals (in humans and animals they have perceived through the senses thanks to complex organizations of nerve cells that have different tasks such as the perception of the environment or the recognition of stimuli). They are processed in information through an impressive number of neurons (which represent the computing capacity) interconnected with each other in a non-linear and variable structure in response to those external data and stimuli themselves. It is in this perspective that we speak of a mathematical-computer model. In the same way, ANNs are non-linear structures of statistical data organized as modeling tools: they receive external signals on a layer of nodes (which represents the processing unit, the processor). Each of these “input nodes” is connected to various internal nodes of the network, which, typically, are organized at multiple levels so that each node can process the signals received by transmitting the result of its processing (therefore more advanced, detailed information) to the subsequent levels. In principle, NNs are made up of three layers (which, however, can involve thousands of neurons and tens of thousands of connections): (1) the input layer (I-Input): it is the one that has the task of receiving and processing the input signals adapting them to the requests of the network neurons; (2) the so-called H-hidden layer (hidden layer): it is the one in charge of the actual processing process (and can also be structured with multiple column-levels of neurons); (3) the output layer (O-Output): here, the results of the processing of layer H are collected and are adapted to the requests of the next block-level of the NN. For this process to perform, it is necessary to train the NNs, that is, make them learn how to behave when an engineering problem will be solved, such as the recognition of a human being by analyzing the images. The learning theme is connected to ML, understood as algorithms that use mathematical-computational methods to learn information from experience.

These are the leading models in use today:

- Supervised learning: the algorithm provides both data sets as input and information relating to the desired results with the aim that the network identifies a general rule that connects the input data with the output data; in other words, examples of inputs and outputs are provided so that the system learns the link between them and extrapolates a rule that can be used for other similar tasks;
- unsupervised learning: only data sets are supplied to the system without any indication of the desired result. The purpose of this second learning method is to “go back” to hidden schemes and models, that is to identify a logical structure in the inputs without these being previously labeled;
- reinforcement learning: in this case, the system must interact with a dynamic environment (which allows it to have input data) and achieve a goal, also learning from the errors (identified by “punishments”). A learning routine determines the behavior of the system based on reward and punishment;
- semi-supervised learning: it is a hybrid model where the computer is provided with an incomplete data set for training/learning; some of these inputs are “equipped” with the respective examples of outputs (as in supervised learning), others are without them (as in unsupervised learning). The goal is always the same: to identify rules and functions for solving problems, as well as models and data structures useful for achieving specific objectives.

**Fig. 2:**
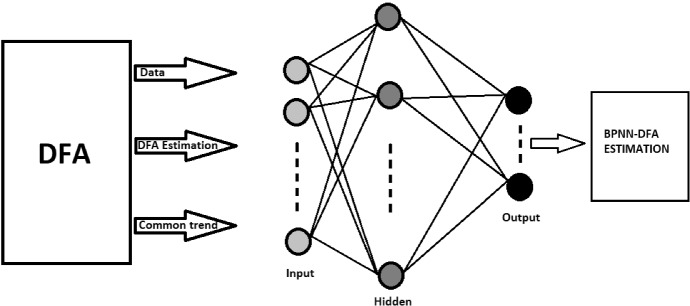
The BPNN-DF process. Source: our elaborations in YaD

Now, once we understand the functioning of a generic ANN, we try to adapt this process to the DF model used in the time-series analyses previously shown. This procedure is very complicated for computer programming and ML theory. The NN algorithm must elaborate on a dataset containing a time series and different countries in the context of the DF model. In other words, we need to verify the dynamic effect on each target country, which represents the same inputs in the model. The logic of NN would not allow this type of analysis. The target represents *n* −  1 NN input, which is generated after numerous factorial combinations between the same inputs. Therefore, we use the contribution that meteorological science has made to the construction of NNs. Following the indications of Rumelhart et al. (1986), we adopt a hybrid process of back-propagation neural networks (BPNN) connected to a DF model. The basic idea is to use the DF model to extract the main factors of each country, and analyze the common trend through a BPNN. It is able to model a non-linear relationship between the main factors of the DF model and its estimates. Finally, we get a model as a sum of two different algorithms. Figure 2 shows the analysis process.

As we can see from Fig. 2, the BPNN model presents a feed-forward architecture. Its framework consists of an input layer, one or more hidden layers and one target (output) layer. The goal of the supervised BPNN in the training state is to minimize the error. So, we first developed an algorithm in Java capable of adapting the results of the time-series DF model to a NN algorithm. At the same time, we expanded the dataset through the logarithmic and quadratic transformations (*Ln* and *S*). In this way, in our algorithm of a classic NN, it has been possible to elaborate 7,854,739,200 × 11^3^ different combinations between inputs of the NN—compared to *n* targets—and inputs of the Java algorithm of the DF model. Afterwards, we proceed to generate 21 different NNs for each combination of targets by processing the Java algorithm in Oryx. The obtained results generate a single NN process that uses the same procedure for image processing. Through this procedure, we can be able to overlap all the NNs in a single grade network that synthesizes the Fig. 2. For more details, see the Oryx results and Java procedures provided in the Appendix.

**Fig. 3:**
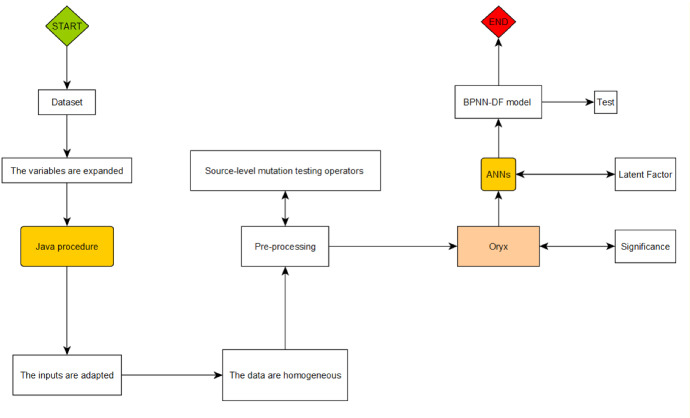
ML flowchart. Source: our elaborations in YaD

The analysis process is constructed in Fig. 3. It is very complex, and it is a novelty for the literature. The complexity of the flow chart lies in the combination of two programming software. They have different languages, and we had to rewrite the algorithms adapting them from Java to Oryx in a recombination process. The result, however, is intriguing. As we can observe from the flowchart, the computational power of Java has allowed an excellent compression of observations that have proved to be homogeneous. The *Source-level mutation testing* process is instead generated through a test-reset effect between Java and Oryx. In other words, the first were intended as input for the second, and vice versa. This analysis yielded a remarkable data mutation test. Finally, from the flow diagram, we can also see how the Oryx software has created a latent factor within an algorithm transcribed in Java. It followed the link of the Poisson algorithms with a Decoding error of less than 5%.

Regarding the construction of the latent factor, we followed the Hofmann (2001) mathematical process:

17 $$ x_{k,z} = u_{k} v_{z} , \forall k,z. $$

Then we use a Markov matrix on (17):

18 $$ X = UV \;{\text{with}} \;U = \left( {u_{1} \ldots u_{M} } \right)V^{t} = \left( {v_{1} \ldots v_{n} } \right), $$

19 $$ Y = R \cdot X = R \cdot \left( {UV} \right), $$

where *R* is made up of 0 with latent ratings, and 1 where ratings are present.

To estimate the latent factor, we solve the following writing:

20 $$ \mathop {\min }\limits_{U,V} Y - R \cdot (UV) + \delta (U^{2} + V^{2} ). $$

Thus, the Candes and Tao (2010) approach can be used:

21 $$ \mathop {\min }\limits_{x} Y - R \cdot X^{2} + \delta X_{NN} . $$

**Fig. 4:**
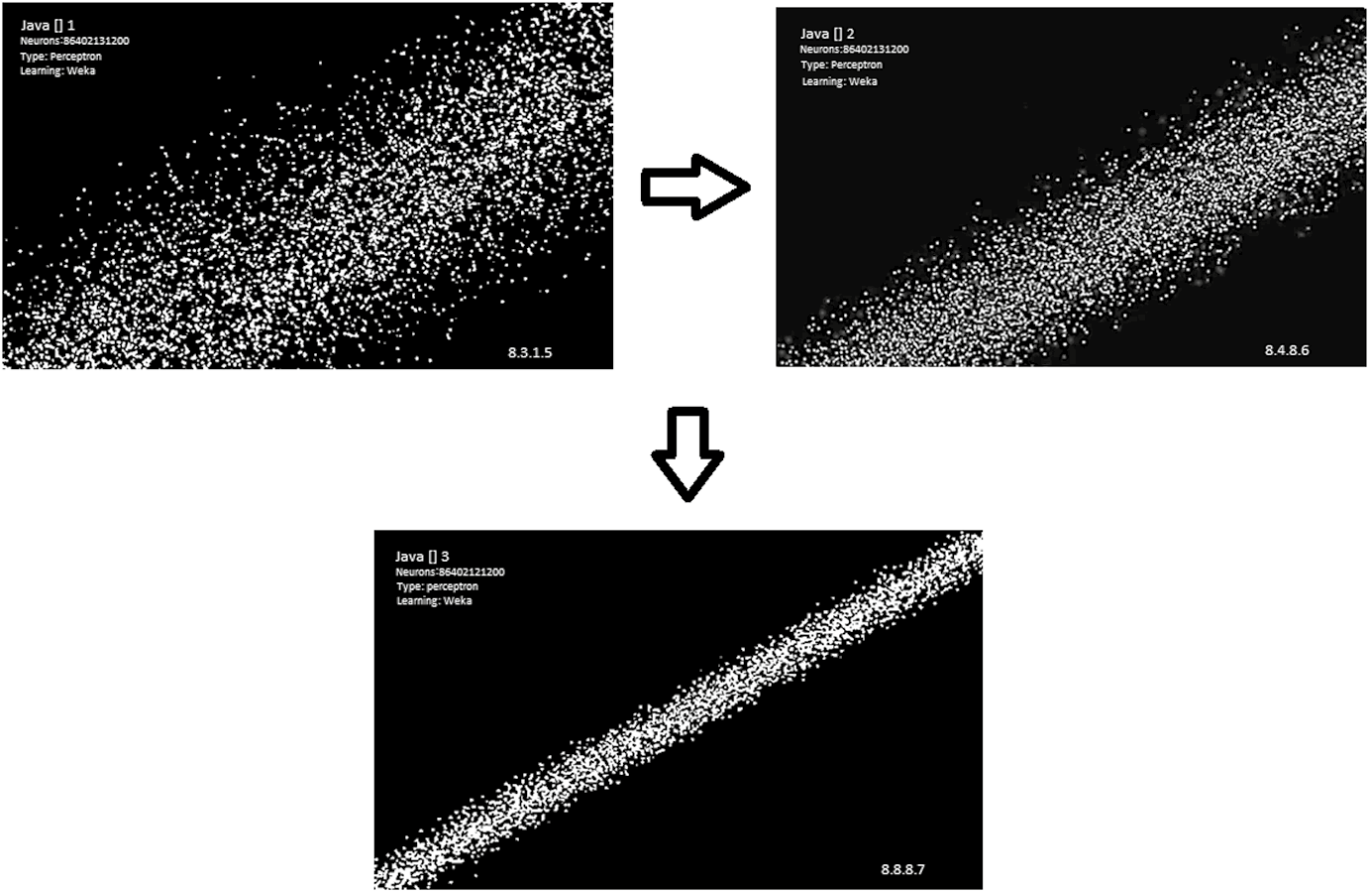
Double imaging learning mutation. Source: our elaborations in Java

As anticipated, the first analysis process aims to adapt the time-series dataset. In particular, we modified the combination of inputs that represented the same outputs of the analysis. The goal here is to prepare a homogeneous data flow capable of generating numerous targets equal to the factorial combination of the same inputs (Fig. 4).

As we can see from Fig. 4, the analysis of the image processing proceeds towards a dispersion reduction of the single neurons that can be observed by the numerous white dots. After having been duplicated through an input-target-input-target logic, they were aggregated towards a single analysis system that can be observed in the last image bearing the word *Java [] 3*.

**Fig. 5:**
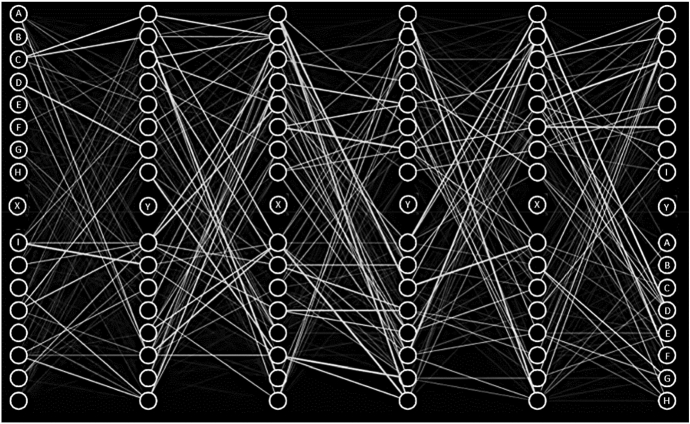
Input-target-input-target imaging combination. Source: our elaborations in Java

Figure 5 clearly shows the reason for the programming process. The inputs, represented by the letter *A* to *I* with the addition of *X* and *Y*, are the countries of econometric analysis. We can observe that they are also present in the last phase to the right of the continuous and dynamic NN (the image represents one of the *n* switching on and off steps of the NNs). This result is due to the presence of more countries for more data, which generally affects the dynamic cycle. The whole process used is present for each level, objective and operation in Table 2.

**Table 2:** Source-level mutation testing operators for ML system

Type	Level	Target	Operation
DR	Global	Data	Duplicates specific types of data
LE	Global	Data	Falsify specific results of data
DM	Global	Data	Remove specific type of data
DF	Local	Data	Shuffle specific type of data
NP	Local	Data	Add noise to specific type of data
LA	Global	Prog	Add a layer
AFR	Global	Prog	Remove activation functions

*DR* data repetition, *LE* label error, *DM* data missing, *DF* data shuffle, *NP* noise perturbation, *LA* layer addition, *AFR* activation functions remove

Once the data was harmonized in Java, we transferred the new data process into the Oryx dataset capable of analyzing *n* number of targets concerning *n* + 1 input where *n* represents the extended targets (Table 3).

**Table 3:**
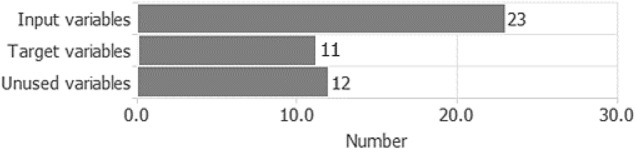
Variables bars chart. Source: our elaborations in Oryx

**Fig. 6:**
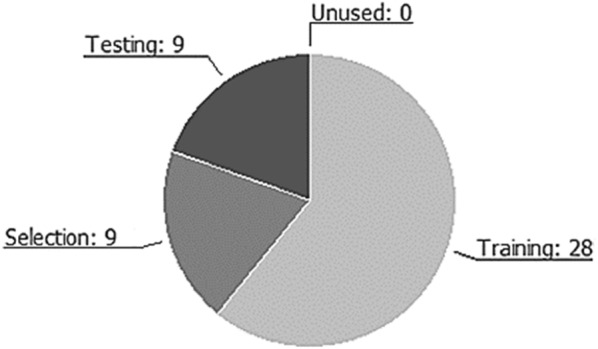
Instances pie chart. Source: our elaborations in Oryx

Table 3 shows the use of variables in Oryx processing. In particular, we can note that the input variables used are 23. They are the result of the variables that represent the individual countries and their logarithmic transform. The quadratic transforms, however, have not been used automatically by the system and therefore they are present in the unused variables item (12). Target variables are the most interesting part of the table. Compared to a generic neural network process, they are numerous (11), resulting in a multivariate approach > 3. This value (target) shows the success of the Java adaptation of the dataset, which needed, in a dynamic model, to consider the effects of the same inputs as targets. In other words, we can imagine that the Oryx algorithm was able to generate 11 different NN processes in a single operation. Subsequently, we observed the behavior of the instances in the dataset through the pie chart in Fig. 6.

**Fig. 7:**
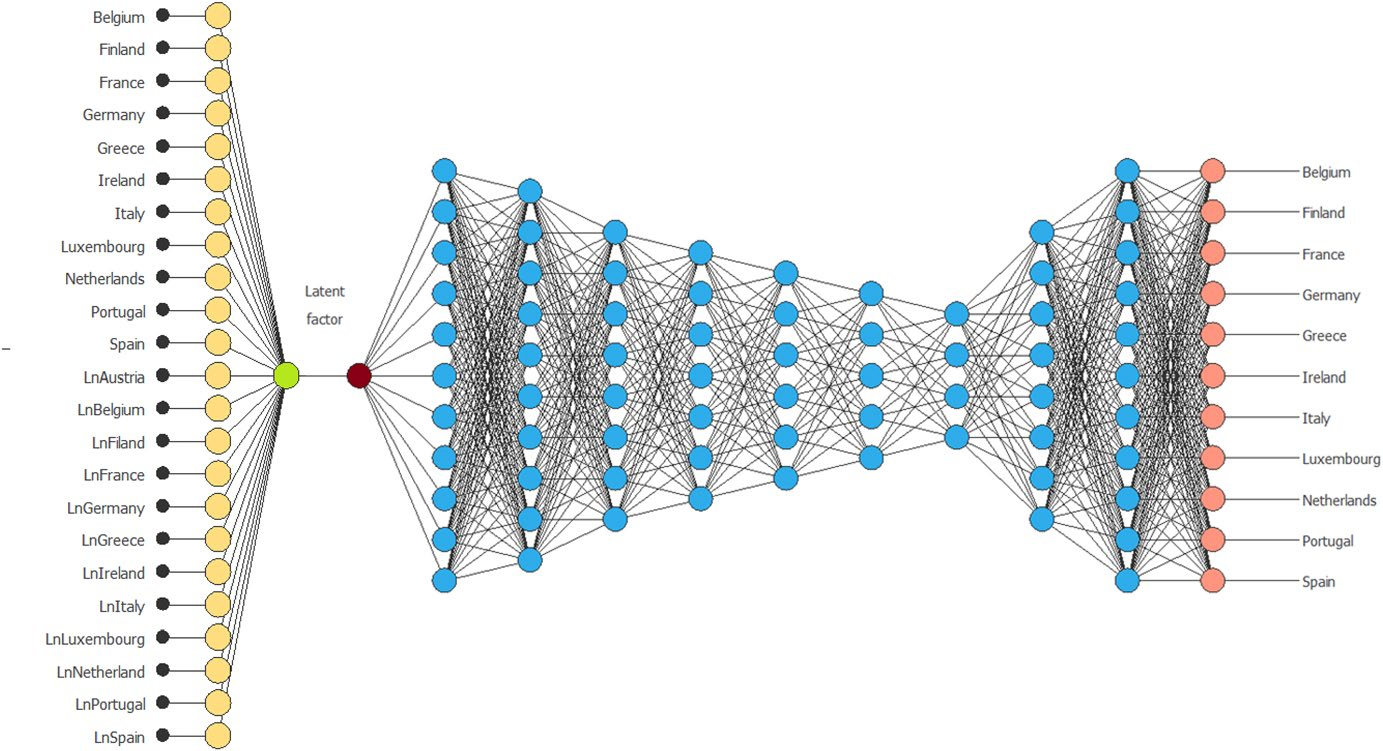
BPNN-DF model. Source: our elaborations in Oryx

As we can see from Fig. 6, the instances work in a ML model as a sort of samples or points. They are able to behave, in the analysis, in a way that manages the process. We have obtained a total number of instances equal to 46. The number of training instances is 28 (60.86%). This result shows how, compared to a predetermined number of potential designs of the project, the model compared twenty-eight different architectures. Concerning the number of selection instances, we obtained a value of 9 (19.56%). The nine instances have chosen the best process of ANNs that can best generalize the algorithm. The number of test instances is 9 (19.56%). These instances generate numerous training models, finally choosing the one that works best. The value obtained allows us to continue the analysis optimally. Finally, the number of unused instances is 0 (0%). This result confirms the high reliability of the data created by the Java algorithm. The transposition in Oryx did not generate abnormal values in the data that can cause NN to malfunction. After observing the behaviour of the datasets concerning the processing in ML of our algorithm, we can analyze the result of the BPNNs in Fig. 7.

The BPNN-DF model graph in Fig. 7 contains four scaling levels, one NN and eleven non-scalable levels.^4^ The yellow circles represent scaling neurons, the blue circles perceptron neurons, the dark red circle is the latent factor and the red circles are the unscaling neurons. The number of inputs is twenty-three, and the number of outputs is 11. The complexity, represented by the number of hidden neurons, is 11:10:8:7:6:5:4:8:11. The execution time for each input-target combination was 175 s on average, with a peak of 340 s in possible combinations of logarithmic and target inputs. The distribution of NN is a variable step process. This effect is clear from the 4:8:11 complexity. Each neuron is interconnected with each other and between the perceptrons of the subsequent networks. The inputs are connected to the NN process and, therefore, to the targets through the latent factor. It has been positioned in this connection mode to linearize this effect homogeneously to each target. The transformation for training from dynamic to static (carried out via Java) and its propagation again in the form of a dynamic network has generated numerous input-target equations. The pairs of the same input-target, which are affected by the entire combinations of the perceptrons, have shown some expert variations in Table 4.

**Table 4:** BPNN-DF model predicted target expression. Source: our elaborations in Java and Oryx

BPNN-DFAm	Countries	Results
y_1_1 = Logistic	Belgium	6.8
y_2_2 = Logistic	Finland	14.2
y_3_3 = Logistic	France	4.9
y_4_4 = Logistic	Germany	− 15.0
y_5_5 = Logistic	Greece	9.0
y_6_6 = Logistic	Ireland	− 17.5
y_7_7 = Logistic	Italy	8.7
y_8_8 = Logistic	Luxembourg	− 15.5
y_9_9 = Logistic	Netherlands	− 13.5
y_10_10 = Logistic	Portugal	7.6
y_11_11 = Logistic	Spain	9.4

The results in Table 4 are the sequence of a dynamic process whose variations can be classified as the economic cycle of the EMU in the period 1970–2014. These results represent those reported in Table 1 concerning the beta coefficient. On a predictive level, we can note that the economic cycle has a significantly positive impact on the revenues ratios for seven economies. Except for the case of Austria, which was the variable automatically eliminated by the connection process of the rectal networks, all the variables expressed a predictive value of the target. It represents the result of the combinations between input and the respective target. Unlike the econometric model, the results do not show a level of significance. Their value is considered concerning a positive or negative predictive factor. Therefore, observing the empirical findings in Table 4, we note that Germany, Luxembourg, Netherlands and Ireland show negative values; thus, the economic cycle has not had a positive impact on their revenues ratios. These results confirm, except for Luxembourg, those of the DF model. The results of the confusion matrix in Table 5 confirms the findings obtained through BPNN-DF model.

**Table 5:** Confusion matrix. Source: our elaborations in Java and Oryx

	Predicted positive	Predicted negative
Real positive	14,728	581
Real negative	674	12,101
Classification Accuracy	95.54%	
Error rate	4.46%	
Sensitivity	96.20%	
Specificity	94.72%	
False positive	5.27%	

It is a specific layout that allows us to view the performance of the algorithm. It plays the role of being a particular type of contingency table, with two “real” and “predicted” dimensions, and an identical set of classes in both dimensions. The predicted values, compared to the actual positive values, cause a change in the target 96.20 times every 100 combinations between the inputs made. Therefore, compared to the actual positive values, there is only a 3.8% probability of being able to choose a different Targets than that obtained in the analysis of BPNN-DF model. Besides, the confusion matrix was tested with 5 experiments. *Classification Accuracy* measures the percentage of exact predictions out of total instances. It is the inverse of the error rate, and it ranges from 0 (worst) to 1 (best). We have transformed the value into a percentage, obtaining a result of 95.54%. The *Error rate* measures the percentage of error of the predicted value on the total of the instances. It ranges from 0 (worst) to 1 (best). We have transformed the value into a percentage obtaining a result of 4.46%. *Sensitivity* is the percentage of correct positive predictions out of the total of positive instances. It ranges from 0 (worst) to 1 (best). Also, in this case, the value has been transformed into percentages and is very high, equal to 96.20%. *Specificity*, on the other hand, represents the percentage of predicted negative corrected on the total of negative instances. Again, it ranges from 0 to 1 with the highest best value. We, in percentage terms, have obtained a value of 94.72%. Finally, *False Positive* is a rate that measures the percentage of incorrect positive predictions on the total of negative instances. It varies from 0 (best) to 1 (worst) compared to the others indicated. Also, in this case, the result was excellent, with a value of 5.27%. Therefore, we can affirm that the analysis of the confusion matrix in the table (j) correlated by the five tests carried out confirms the goodness of the results obtained by the NN.

**Fig. 8:**
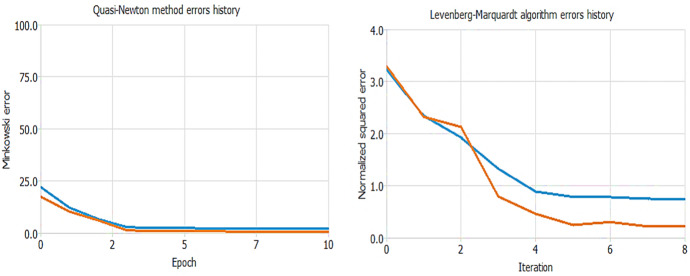
Training Process test. Source: our elaborations in Oryx and NeuralAd

Finally, we test the training process. We follow the belief that the best training strategy is the one that allows the best possible loss in the NN. We use two different tools: the Quasi-Newton method and the Levenberg–Marquardt algorithm in Fig. 8.

Concerning the Quasi-Newton method graph, it calculates an inverse Hessian approximation at each iteration of the algorithm, using only gradient information. In the diagram, we can observe the training and selection errors in each iteration. The blue line represents the training error, and the orange line represents the selection error. The initial value of the training error is 22.0876, and the final value after 10 epochs is 2.09872. The initial value of the selection error is 17.4587, and the final value after 10 epochs is 0.519827. Therefore, since the lines drop rapidly as the epochs increase, the first test is correct. The Levenberg–Marquardt algorithm, on the other hand, is used here for training, designed to approach second-order training speed without having to calculate the Hessian matrix. The figure shows that the training and selection errors in each epoch are reduced to the value of 0.217589.

The latent factor, in reality, could be the result of various forces that pushed in the same direction. First of all, the similarity of the economic cycles of the EMU countries starting from the Werner Plan (European Commission 1990; Marelli et al. 2019). Since the introduction of the euro, both β-convergence—which measures whether countries with lower GDP per capita grow faster than those with higher GDP per capita (so-called “catching up effect”)—and *σ*-convergence—which measures the decline in dispersion of countries’ GDP per capita—have improved among EU members, but this improvement has slowed compared to the two prior decades (Cabrillac 2019). However, the Great Financial Crisis actually stopped convergence among EU countries and regions (Caldera Sánchez 2018).

Furthermore, the structure of the European tax systems since 1970 has generally moved towards greater central taxation and a revenue structure that affects income rather than wealth.

These factors are inextricably linked and feed themselves, amplifying the effect of an underlying force capable of leading to a general convergence of public revenues of the member countries of the EMU.

If there is a convergence of shocks, they tend to become more symmetrical, and an approximation of the tax systems and the revenue structure increases the economic integration of the area.

Hence, the importance of a latent factor in the revenue is crucial, as it favors the possibility of a political union, however it is not yet ready due to cultural reasons, language, and religion.

Ultimately, the latent factor that emerges from our empirical analyses may be also due to the common willingness to secure public accounts under the Maastricht Treaty, which is consistent with the explanation in Albanese and Modica (2010) and Pan and Wang (2012).

## Visibility of a Latent Factor: Analysis and Process

Latent factor experiments have been widely exploited in recent years in any experimental and non-experimental science. However, standard estimation techniques assumed an unreal process. In other words, the analysis simulated the organization of data in time series and discrepancies, generating a case of a latent factor over time. The transition from a simulation, almost theoretical-empirical forced, to a simulation based on the non-visible data’s reality is expansive. In general, deep learning or machine learning has solved this problem by bringing attention to the identification of latent data. Through complex techniques of “collaborative algorithm filtering”, it is possible to highlight the latent factor of one or more-time series.

Nevertheless, the analysis requires the writing of a supervised algorithm with n layers. The complexity of this operation derives from instructing each command string in the search for an invisible value that can influence the visible data. In other words, the algorithm has to overcome a paradox: to make visible what is not.

**Fig. 9:**
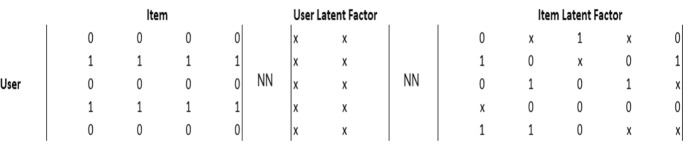
NN binary latent factor. Source: our elaborations in YeD

To overcome the paradox that characterizes the latent factor, we propose an original idea that has never been dealt with in literature. In particular, we start from the basic assumption that every data present in a machine learning process is nothing more than an infinite series of binary combinations between 0 and 1. Therefore, both the inputs and the outputs of a neural network represent the result of signals between 0 and 1. So, if the previous condition is true, it means that the presence of a latent factor could represent a time gap between the binary signal. Therefore, this factor is also present in the output, which is the result of the binary combinations of the inputs influenced by the latent factor. We have represented this idea in the underlying body (Fig. 9).

As we can see from Fig. 1, we are faced with a large matrix. It can be represented as a product of the user’s latent factors matrix (User) and the latent factors matrix. Therefore, it represents a product of two matrices, which we can solve with a matrix factorization.

Therefore, the mathematical formulation—to which we apply the concept of non-negativity—can be written as:

22 $$ K = JY. $$

Since the NN, as shown in Fig. 7, has multiple levels, we can have:

23 $$ K = J_{1} J_{n} Y, $$

where $J_{1} J_{n}$ are the levels about the latent factors, $Y$ is the signal in the NN.

Now, using the mathematical technique of non-negativity in a binary context, we obtain:

24 $$ \rho = B \cdot K, $$

where $B$ is the binary matrix of 1’s and 0’s and $\rho$ the acquired ratings matrix.

We use (23) and (24):

25 $$ \rho = B \cdot K = B \cdot (J_{1} J_{n} Y). $$

Now we can solve the previous writing:

26 $$ \mathop {\min }\limits_{{J_{1} J_{n} Y,K}} \frac{1}{2}\left\| {\rho - B \cdot (J_{1} J_{n} Y)} \right\|_{q}^{2} \;{\text{with}}\;J_{1} J_{n} Y \ge 0. $$

To solve (26) we use a complex process called projected descending gradient (PDG). Thus, we can assume that:

27 $$ K^{0} , J_{1}^{0} , J_{n}^{0} , Y^{0} \;{\text{with}}\;u = 1,2 \ldots n, $$

28 $$ \overline{{K^{k} }} = K^{k} - \delta \left( {B^{t} \cdot \left( {B \cdot K^{k} - \rho } \right)} \right), $$

29 $$ (K^{k + 1} , J_{1}^{k + 1} ,J_{n}^{k + 1} , Y^{k + 1} ) \to \mathop {\min }\limits_{{K,J_{1} J_{n} ,Y}} \left\| {\overline{{K^{k} }} - K} \right\|_{q}^{2} . $$

We trigger the algorithm of the descending gradient limited by the projection of itself:

30 $$ J_{1}^{k + 1} = L_{ + } (J_{1}^{k} - \left( {J_{1}^{k} J_{n}^{k} Y^{k} - K^{k + 1} } \right)(J_{1}^{k} J_{n}^{k} Y^{k} K^{k} )^{!} , $$

31 $$ J_{n}^{k + 1} = L_{ + } (J_{n}^{k} - \left( {J_{1}^{k} )^{!} (J_{1}^{k} J_{n}^{k} Y^{k} - K^{k + 1} } \right)(J_{n + 1}^{k} K^{k} ))^{!} , $$

32 $$ Y^{k + 1} = L_{ + } \left( {Y^{k} } \right)\left( {(J_{1}^{k} J_{n}^{k} )^{!} \left( {J_{1}^{k} J_{n}^{k} Y^{k} - K^{k} } \right)} \right). $$

**Fig. 10:**
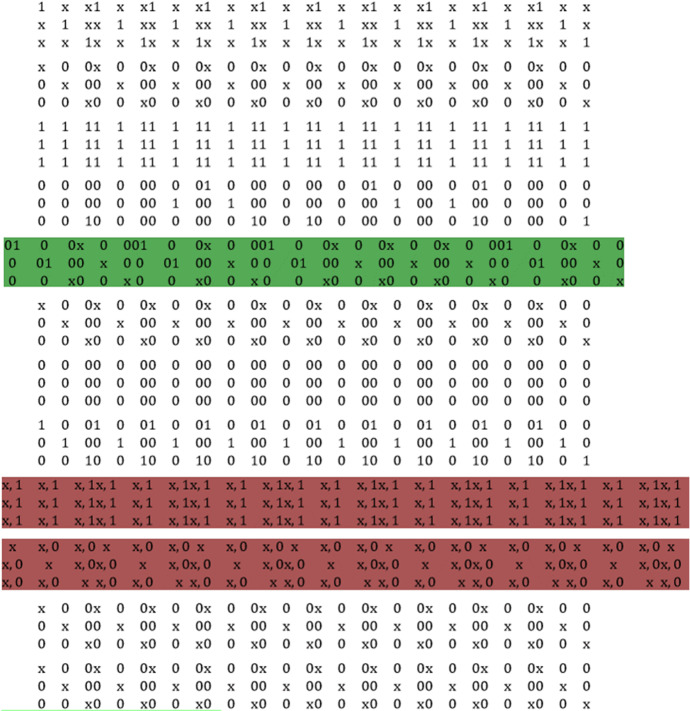
Binary strings process. Source: our elaborations in Java

**Fig. 11:**
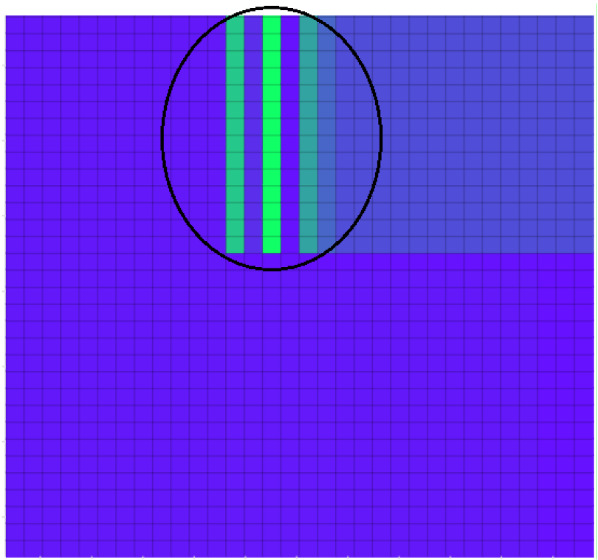
Latent factor in binary operations. Source: our elaborations in Java and Oryx

The prospect that can be observed in Fig. 10 is the result of 12 binary strings in the algorithm. They result from a synthesis of over 7450 strings executed through the projected drop gradient settings. We have chosen to present these 12 strings because they are a very good sample of the whole binary process. Each string may or may not have a linear binary combination. As we can see, some, in addition to the standard compartment 0 and 1, there is the latent factor. We instructed the algorithm to highlight the latent factor as a gap in the timeline between 0 and 1. Next, we implemented the command through forced supervision of the algorithm. This procedure allowed the software to highlight the latent factor between 0 and 1 with an (*x*). Non-highlighted binary strings are inconclusive for the analysis. They represent the standard sequencing between 0 and 1. Although the variable *x* is present in these command lines, it is not a latent factor. In fact, as can be easily observed, the binary factor is only reproduced to the following string (see string 1 and 2), where the value 0 exists in a string and the value 1 in the subsequent one, or vice versa. In this case, the variable *x* is not a latent factor but only the normal computational process of software processing. The situation of the string marked in green is different. In it, there is a normal binary process between 0 and 1. Between them the variable *x* recurs many times. In this case, it represents a time lag (even if of one thousand thousandths of a second) between the execution of the command 0 and 1. In our case, it is the time lag in the NN. Finally, the strings highlighted in red represent an information overload between the binary session and the need to estimate the latent factor. Therefore, these are errors that have been promptly eliminated from the system. The graphic result of the 12 strings is represented by Fig. 11.

**Fig. 12:**
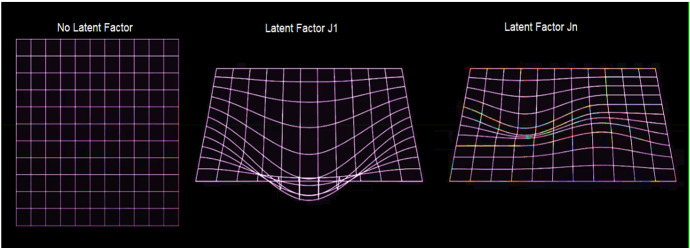
The latent factor visible in the dataset. Source: our elaborations in Java and Oryx

**Fig. 13:**
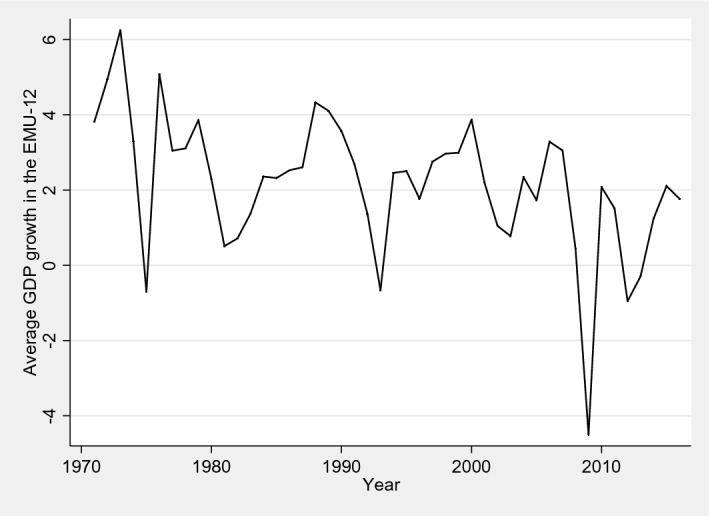
Average GDP growth in the EMU countries (12 EMU countries, 1970–2014). Sources: our elaborations on AMECO and PWT data.

Figure 11 shows a binary sequence box. Usually, it should be homogeneous in colour and, therefore, all blue. However, it has three processing biases. The first is at level 13 × 14; the second is highlighted in 15 × 14; the third at level 17 × 14. These distortions, highlighted in green, are the latent factor from *J* to *J*_*1*_, reaching up to *J*_*n*_. *J*_*1*_ is the maximum signal of the latent factor and is highlighted in the figure with the brightest green color. Next, we have chosen to isolate the three distortions of the binary process in Fig. 11. In particular, we have instructed the algorithm using the same procedure used in seismology to isolate *P* and *S* waves. The result can be seen in Fig. 12.

Figure 12 represents the three-dimensional processing of the latent factor in the dataset. It is a Java projection of the result obtained in Fig. 11. The first graph represents the blue part of Fig. 11. In it, there is no time distortion between the binary factor 0 and 1. Therefore, the data are distributed in an orderly manner on a plane. The second graph, on the other hand, clearly shows the presence of the latent factor. It coincides with the bright green part of Fig. 11. We can note how it respects Eq. (9). When the learner gradient analyzes the time gap between 0 and 1, the plane undergoes a distortion in three dimensions. The convexity of the figure is the latent factor in the binary analysis. Subsequently, *J*_*n*_’s factor in Eq. (10) comes into play, which harmonizes the time distortion between 0 and 1 in subsequent binary operations. This graphical representation is the third graph in Fig. 12. At the level of computer programming, the result obtained from Figs. 11 and 12 represent the first empirical demonstration of the latent factor’s presence.

## Concluding Remarks and Policy Implications

The aim of this paper is to show that an unobserved common factor drives the co-movement of government revenues in the EMU. We estimate a common component in the series of government revenues after partialling out the effect of EMU business cycle by introducing explicitly this variable in the empirical analysis. To the best of our knowledge this is the first study that attempts to inspect the presence of an unobserved common factor for public revenues. In addition, this is the first study that applies a NNs approach to the topic of public revenues in a monetary union. We have found that this common component has a significant impact on government revenues in nine out of twelve countries (the exceptions being represented by Belgium, Greece, and Italy). Meanwhile, there exists a systematically positive relationship between EMU business cycle and the revenues ratios for nine economies (all but Germany, Ireland, and Netherlands). We highlight this common pattern in a dynamic factor model, and argue that a satisfactory explanation for it would be desirable. In fact, as the factor is unobservable and we can only extract an estimate of it, it is difficult to agree on what it represents. This evidence is consistent with the findings in Albanese and Modica (2010) regarding the co-movement of public spending in the G7, and the results by Pan and Wang (2012) on the co-movement of government debt in the EMU. Furthermore, our empirical results confirm the profound differences which exist between the various members of the EMU regarding public finances, as pointed out by Brady and Magazzino (2018a).

The economic and financial crisis appears to have set in motion a process of deepened European economic policy integration. The co-movement of federal revenue in the EMU depends on the fiscal policies set aside by the member countries. The analytical factor analysis above indicates the direct impact of the policies guiding the EMU institution. The industrial sector has a significant effect on monetary policy. Furthermore, it is clear that when favorable fiscal policies are implemented, then the systems tend to create a pleasant business boom environment. Industries tend to invest more, thus creating employment. The population will have adequate funds to spend and invest. Labour movements get more flexible and significant within the EMU states. This scenario minimizes the shock factors that negatively inflict and suppress the revenue flows.

We have tested the results obtained from time-series model through a procedure of ANNs in a dynamic model of the DF type. This procedure has never been used in a socio-economic research. Therefore, we have used the same procedure to study in a NN context the dynamic effects of the economic cycle on the revenues of the countries taken into consideration by the time-series analysis. The results obtained, after carrying out seven different tests, confirmed the DFM ones. Notwithstanding, as explained in Albanese and Modica (2010), providing an interpretation of a common factor is a hard task, because the factor is unobservable; thus, it is difficult to agree on what it represents. Following De Grauwe (2018) and Baldwin and Wyplosz (2019), we can argument that this paper supplies a further proof of the existence of a “deep variable” that connects the economies of the EMU. We have argued that the latent factor that emerges from the two different empirical approaches here used could be of a composite nature, being the result of both the more general convergence of the economic cycles of the countries in the area and the increasingly better tuned tax structure.

Previous latent factor studies used simple software implementations to execute necessary factor analysis commands. For example, the authors implemented some STATA executions through the “factormat and sem” package. This result coincides with the assumption about the stationarity of the operator. It executes a series of commands in an environment with results predetermined by the software. Therefore, if in the implementation package a series of data subject to factor analysis decides that the assumption of the latent factor exists, it will be considered as such by the researcher. However, this result is not an image of the real latent factor. It becomes so only after an accurate interpretation by the researcher. Even surpassing the Metropolis–Hastings algorithm approach (Cai 2010), our estimation process used a complex mechanism. It is the real observation of the latent factor in a binary environment. Therefore, it represents the result of writing an algorithm that does not “extract” a hypothetical latent factor, but observes the real presence of the latent factor. The extraction process, typical of inferential analysis, has the limit of damaging the series of data being estimated, invalidating—at least in our opinion—the entire analysis process. Using a supervised-unsupervised approach, we were able to verify the presence, even in three dimensions, of the latent factor within our dataset.

The current fiscal policies put in place do not cater for emergencies such as financial crisis, unemployment, recession, immigration. All these are variables that need more significant concern (De Grauwe and Ji 2013). Moreover, most of the countries only cater to the current times, thus neglecting the future. A more stable and prosperous future in terms of growth and development of the EMU region would only take place if the policies put in place could cater to critical moments. Policies that encourage government subsidies such as tax alleviation, research, industrialization, education, and government expenditure would allow the EMU member countries see an increase in GDP that will enhance sufficient public revenue flows. Most of the policies within the EMU institutions are out of date and are not consistent with the needs underlined by the current trends in economic growth within the region. The current fiscal policies hinder sufficient public revenue flows within the entire region. Each of the member countries is allowed to play an integral role for coordination purposes. Although in the persistence of some structural differences in the economy, the autonomous fiscal policies at the national level is now the only tool left to countries to solve these differences.

EMU faces a significant challenge as it does not have central control over fiscal policymaking. The organization heavily relies on the new policies that were set during its formation; hence the need to allocate more power to the organization so that it can design fiscal policies which guide the entire EMU region. The current budgetary systems require a significant overhaul where policymakers need to dialogue and create friendly fiscal policies that will encourage public revenue flow. The current policies only favour individual countries and the result is to give rise to dominant countries that seem to control the EMU economy (Rey 2018). The euro creation was to enhance sustainability, balance in the marketplace, and also encourage trade links between the member countries. The advantage is that the euro is a stable currency and has spurred trade between member countries, thus providing a productive trading environment and also significant public revenue flows. However, the member countries need to contribute promoting fiscal policies that favor the entire region. Creating strategies that would encourage multilateral trade would see an increase in public revenue beyond sovereign borders. The plans that need to be put into place include, policies to open up borders, strategies to encourage multilateral trade, policies aimed to financing industries, research education, and research. Providing the EMU with more power to create centralized fiscal policies would spur up public revenue flows.

At present, we have a very variegated and inconsistent framework of criteria for determining the tax base, as well as the rates in the various EU countries, and this situation is likely to create distortions and discrimination and, consequently, to encourage opportunistic behavior on the part of both States and individual tax payers. To complete the integration, therefore, a different idea of taxation must be gained at European level, also as a tool for gathering the essential financial resources for the development and existence of a community according to fair distribution principles.

The revenue shocks arise from the policies implemented by each sovereign government. If EMU could be given the full mandate of establishing fiscal standard procedures for all, then co-movement of public revenue could substantiate even more. The institution can create the conventional ground policies that would enhance revenue flow and try to establish a balanced regional economic and development regional growth. The analysis has suggested that fiscal policies designed within the EMU and the sovereign states respectively affects positively or negatively the co-movement of public revenue.

EMU countries experienced a drop in the speed of real convergence after 2008, and converge at a lower speed than the EU. As a consequence, a specific budget for EMU would be important to provide adjustment mechanisms after large shocks Marelli et al. (2019). This paper, highlighting the presence of a latent factor on public revenues for EMU countries, helps to provide empirical evidence in favour of a centralized budget at the European level and the completion of the monetary union, which is currently incomplete.

Further analyses should be conducted in order to better clarify the latent factor evidenced by this study, also using alternative empirical approach to complement those used here (i.e., Panel VAR or Global VAR). Moreover, it would be interesting to consider at least two sub-samples: 1970–1989 (high inflation, pre-core integration period), and 1990–2014 (integration process).

## Appendix

See Table 6 and Fig. 13.

**Table 6:** Descriptive statistics (12 EMU countries, 1970–2014). Sources: our calculations on AMECO and PWT data

Country	Variable	Mean	Median	Standard Deviation	Skewness	Kurtosis	IQR	CV
Austria	R	40.0200	40.5375	2.6078	− 0.8153	2.8437	3.1660	0.0652
	RGDPE	3.1087	3.2383	2.2457	− 0.3328	2.0607	3.5051	0.7224
Belgium	R	41.7513	42.6830	2.8613	− 1.6594	4.9835	2.4900	0.0685
	RGDPE	2.6188	3.2737	3.0237	− 0.8354	3.7876	3.3155	1.1546
Finland	R	40.4732	41.6810	3.9703	− 0.6768	2.3519	6.3480	0.0981
	RGDPE	2.6328	1.8437	3.9713	− 0.3891	3.4580	5.3504	1.5084
France	R	40.6963	41.8910	3.3762	− 1.0454	3.1305	3.2780	0.0830
	RGDPE	2.5309	2.7646	2.6481	− 0.5042	2.9902	3.6675	1.0463
Germany	R	35.3905	35.6375	1.1261	− 1.4956	5.6134	1.3400	0.0318
	RGDPE	2.6220	3.6033	2.5719	− 0.6532	3.1594	3.8969	0.9809
Greece	R	26.8880	26.4030	5.5844	− 0.0142	1.8002	9.8810	0.2077
	RGDPE	2.6127	3.1638	4.4456	− 1.0344	3.8616	4.8785	1.7016
Ireland	R	30.2214	30.2260	2.7181	0.0976	2.2795	4.6170	0.0899
	RGDPE	4.7814	4.5962	4.2548	− 0.5021	3.3996	5.5546	0.8899
Italy	R	35.5527	38.6460	6.6718	− 0.5760	1.8798	10.7970	0.1877
	RGDPE	2.6103	2.4250	2.9466	0.0032	2.1973	4.7397	1.1289
Luxembourg	R	34.8001	36.1425	4.0297	− 1.6222	4.8616	3.8200	0.1158
	RGDPE	4.0676	4.0707	5.7811	− 1.3043	6.3717	6.8452	1.4213
Netherlands	R	38.0690	37.6510	2.3717	0.3116	2.1296	4.0530	0.0623
	RGDPE	2.8127	2.8540	2.9825	− 0.4060	2.7945	4.0995	1.0604
Portugal	R	26.6981	28.4140	5.2703	− 0.5120	2.0280	7.8100	0.1974
	RGDPE	3.3461	3.4224	3.6784	− 0.3600	2.8714	5.0379	1.0993
Spain	R	28.2748	31.4815	6.3775	− 0.7981	2.1922	9.8400	0.2256
	RGDPE	3.2861	4.1375	3.6218	− 0.5012	2.6320	5.4230	1.1021

### BPNN-DFAm Results

**Table:**

scaled_LnAustria = (LnAustria − 3.73663)/0.0235146;
scaled_LnBelgium = (LnBelgium − 3.76805)/0.0211127;
scaled_LnFiland = (LnFiland − 3.76807)/0.034786;
scaled_LnFrance = (LnFrance − 3.75568)/0.0291891;
scaled_LnGermany = (LnGermany − 3.56922)/0.0235573;
scaled_LnGreece = 2 × (LnGreece − 3.13301)/(3.60495 − 3.13301) − 1;
scaled_LnIreland = (LnIreland − 3.40559)/0.0931508;
scaled_LnItaly = 2 × (LnItaly − 3.52689)/(3.78358 − 3.52689) − 1;
scaled_LnLuxembourg = (LnLuxembourg − 3.59315)/0.0527819;
scaled_LnNetherland = (LnNetherland − 3.63669)/0.0633769;
scaled_LnPortugal = (LnPortugal − 3.37917)/0.101512;
scaled_LnSpain = (LnSpain − 3.44999)/0.0976439;
scaled_Belgium = (Belgium − 0.475365)/0.337035;
scaled_Filand = 2 × (Filand − 0.0154139)/(0.979206 − 0.0154139) − 1;
scaled_France = (France − 0.398235)/0.289664;
scaled_Germany = 2 × (Germany − 0.0120771)/(0.951103 − 0.0120771) − 1;
scaled_Greece = (Greece − 0.466647)/0.312268;
scaled_Ireland = (Ireland − 0.494393)/0.270379;
scaled_Italy = 2 × (Italy − 0.0444866)/(0.963327 − 0.0444866) − 1;
scaled_Luxembourg = 2 × (Luxembourg − 0.0870418)/(0.943264 − 0.0870418) − 1;
scaled_Netherland = (Netherland − 0.47236)/0.278992;
scaled_Portugal = 2 × (Portugal − 0.0357318)/(0.970819 − 0.0357318) − 1;
scaled_Spain = (Spain − 0.398344)/0.285646;
y_1_1 = Logistic (− 0.640869+ (scaled_LnGreece × 0.688416) + (scaled_LnIreland × 0.970642) + (scaled_LnItaly × 0.554504) + (scaled_LnLuxembourg × 0.814941) + (scaled_LnNetherland × − 0.913696) + (scaled_LnPortugal × − 0.152405) + (scaled_LnSpain × − 0.36261) + (scaled_AustriaS × 0.647461) + (scaled_Belgium × − 0.323242) + (scaled_Filand × − 0.184082) + (scaled_France × 0.501648) + (scaled_Germany × − 0.217773) + (scaled_Greece × 0.984863) + (scaled_Ireland × 0.0707397) + (scaled_Italy × 0.250427) + (scaled_Luxembourg × − 0.156189) + (scaled_Netherland × 0.333069) + (scaled_Portugal × − 0.944092) + (scaled_Spain × − 0.681335));
y_1_2 = Logistic (− 0.875183+ (scaled_LnGreece × − 0.772156) + (scaled_LnIreland × 0.434082) + (scaled_LnItaly × − 0.0648804) + (scaled_LnLuxembourg × − 0.690002) + (scaled_LnNetherland × 0.839355) + (scaled_LnPortugal × 0.425232) + (scaled_LnSpain × 0.738708) + (scaled_AustriaS × 0.806519) + (scaled_Belgium × − 0.0866089) + (scaled_Filand × 0.656982) + (scaled_France × − 0.751648) + (scaled_Germany × 0.963806) + (scaled_Greece × 0.480225) + (scaled_Ireland × − 0.452576) + (scaled_Italy × − 0.316711) + (scaled_Luxembourg × − 0.296326) + (scaled_Netherland × 0.0372314) + (scaled_Portugal × − 0.183838) + (scaled_Spain × − 0.751099));
y_1_3 = Logistic (0.540771+ (scaled_LnGreece × 0.316711) + (scaled_LnIreland × 0.990784) + (scaled_LnItaly × 0.409302) + (scaled_LnLuxembourg × − 0.387939) + (scaled_LnNetherland × − 0.711975) + (scaled_LnPortugal × 0.42981) + (scaled_LnSpain × − 0.571777) + (scaled_AustriaS × − 0.636108) + (scaled_Belgium × − 0.842529) + (scaled_Filand × 0.793945) + (scaled_France × 0.375122) + (scaled_Germany × 0.533936) + (scaled_Greece × − 0.771729) + (scaled_Ireland × 0.998962) + (scaled_Italy × − 0.972717) + (scaled_Luxembourg × − 0.782837) + (scaled_Netherland × − 0.0648193) + (scaled_Portugal × 0.58136) + (scaled_Spain × − 0.747253));
y_1_4 = Logistic (− 0.335205+ (scaled_LnGreece × − 0.623047) + (scaled_LnIreland × − 0.314331) + (scaled_LnItaly × 0.425232) + (scaled_LnLuxembourg × − 0.920349) + (scaled_LnNetherland × − 0.113342) + (scaled_LnPortugal × − 0.0139771) + (scaled_LnSpain × 0.633728) + (scaled_AustriaS × − 0.667358) + (scaled_Belgium × − 0.128784) + (scaled_Filand × − 0.334656) + (scaled_France × − 0.892761) + (scaled_Germany × 0.1474) + (scaled_Greece × 0.412659) + (scaled_Ireland × 0.00952148) + (scaled_Italy × − 0.608643) + (scaled_Luxembourg × − 0.322937) + (scaled_Netherland × − 0.318542) + (scaled_Portugal × − 0.382019) + (scaled_Spain × − 0.643127));
y_1_5 = Logistic (0.644653+ (scaled_LnGreece × 0.302551) + (scaled_LnIreland × − 0.44458) + (scaled_LnItaly × 0.79541) + (scaled_LnLuxembourg × − 0.725891) + (scaled_LnNetherland × 0.659058) + (scaled_LnPortugal × 0.790283) + (scaled_LnSpain × 0.291443) + (scaled_AustriaS × 0.844604) + (scaled_Belgium × 0.791443) + (scaled_Filand × 0.802856) + (scaled_France × 0.200073) + (scaled_Germany × − 0.248047) + (scaled_Greece × 0.751404) + (scaled_Ireland × − 0.869446) + (scaled_Italy × − 0.167725) + (scaled_Luxembourg × − 0.705017) + (scaled_Netherland × − 0.0516968) + (scaled_Portugal × 0.62854) + (scaled_Spain × 0.373108));
y_1_6 = Logistic (0.246399+ (scaled_LnGreece × − 0.334778) + (scaled_LnIreland × − 0.43396) + (scaled_LnItaly × − 0.531555) + (scaled_LnLuxembourg × − 0.862793) + (scaled_LnNetherland × 0.431763) + (scaled_LnPortugal × − 0.64032) + (scaled_LnSpain × − 0.000427246) + (scaled_AustriaS × − 0.766174) + (scaled_Belgium × 0.794983) + (scaled_Filand × − 0.857361) + (scaled_France × − 0.463684) + (scaled_Germany × − 0.545166) + (scaled_Greece × − 0.826721) + (scaled_Ireland × 0.278381) + (scaled_Italy × − 0.866028) + (scaled_Luxembourg × 0.197876) + (scaled_Netherland × 0.683228) + (scaled_Portugal × − 0.511963) + (scaled_Spain × − 0.584839));
y_1_7 = Logistic (0.0271606+ (scaled_LnGreece × 0.237122) + (scaled_LnIreland × 0.0777588) + (scaled_LnItaly × 0.287476) + (scaled_LnLuxembourg × − 0.490845) + (scaled_LnNetherland × 0.153992) + (scaled_LnPortugal × 0.703247) + (scaled_LnSpain × 0.365173) + (scaled_AustriaS × 0.167847) + (scaled_Belgium × − 0.759094) + (scaled_Filand × − 0.961304) + (scaled_France × 0.214539) + (scaled_Germany × − 0.880859) + (scaled_Greece × − 0.309937) + (scaled_Ireland × − 0.620117) + (scaled_Italy × − 0.667908) + (scaled_Luxembourg × − 0.27832) + (scaled_Netherland × 0.879578) + (scaled_Portugal × 0.22998) + (scaled_Spain × − 0.70459));
y_1_8 = Logistic (− 0.703308+ (scaled_LnGreece × 0.982666) + (scaled_LnIreland × − 0.42981) + (scaled_LnItaly × 0.960022) + (scaled_LnLuxembourg × 0.031189) + (scaled_LnNetherland × 0.00128174) + (scaled_LnPortugal × 0.824524) + (scaled_LnSpain × 0.301392) + (scaled_AustriaS × 0.588562) + (scaled_Belgium × 0.90094) + (scaled_Filand × − 0.538879) + (scaled_France × − 0.767212) + (scaled_Germany × 0.727905) + (scaled_Greece × − 0.688538) + (scaled_Ireland × 0.856812) + (scaled_Italy × − 0.176025) + (scaled_Luxembourg × 0.639038) + (scaled_Netherland × 0.465454) + (scaled_Portugal × 0.90863) + (scaled_Spain × 0.59967));
y_1_9 = Logistic (− 0.458008+ (scaled_LnGreece × − 0.899658) + (scaled_LnIreland × − 0.0301514) + (scaled_LnItaly × − 0.03125) + (scaled_LnLuxembourg × − 0.0150146) + (scaled_LnNetherland × − 0.607544) + (scaled_LnPortugal × − 0.684143) + (scaled_LnSpain × 0.56427) + (scaled_AustriaS × 0.395325) + (scaled_Belgium × − 0.722168) + (scaled_Filand × − 0.81012) + (scaled_France × − 0.284851) + (scaled_Germany × 0.53363) + (scaled_Greece × 0.359253) + (scaled_Ireland × 0.647583) + (scaled_Italy × − 0.338196) + (scaled_Luxembourg × 0.457214) + (scaled_Netherland × − 0.869812) + (scaled_Portugal × − 0.786377) + (scaled_Spain × − 0.18457));
y_1_10 = Logistic (− 0.0245361+ (scaled_LnGreece × 0.976135) + (scaled_LnIreland × − 0.971497) + (scaled_LnItaly × − 0.0300293) + (scaled_LnLuxembourg × − 0.256165) + (scaled_LnNetherland × − 0.0533447) + (scaled_LnPortugal × − 0.193176) + (scaled_LnSpain × 0.694458) + (scaled_AustriaS × 0.00463867) + (scaled_Belgium × − 0.978821) + (scaled_Filand × − 0.467957) + (scaled_France × − 0.886353) + (scaled_Germany × 0.638489) + (scaled_Greece × − 0.581543) + (scaled_Ireland × − 0.474243) + (scaled_Italy × − 0.670044) + (scaled_Luxembourg × 0.135376) + (scaled_Netherland × 0.275024) + (scaled_Portugal × − 0.441162) + (scaled_Spain × − 0.51123));
y_1_11 = Logistic (0.174133+ (scaled_LnGreece × 0.68573) + (scaled_LnIreland × 0.141724) + (scaled_LnItaly × − 0.38739) + (scaled_LnLuxembourg × − 0.25531) + (scaled_LnNetherland × 0.529236) + (scaled_LnPortugal × − 0.812988) + (scaled_LnSpain × − 0.481384) + (scaled_AustriaS × 0.59259) + (scaled_Belgium × − 0.78595) + (scaled_Filand × 0.669922) + (scaled_France × 0.789795) + (scaled_Germany × 0.623108) + (scaled_Greece × 0.578369) + (scaled_Ireland × − 0.755127) + (scaled_Italy × − 0.0439453) + (scaled_Luxembourg × − 0.232666) + (scaled_Netherland × − 0.732849) + (scaled_Portugal × 0.661255) + (scaled_Spain × − 0.284485));
y_1_12 = Logistic (− 0.939514+ (scaled_LnGreece × − 0.907898) + (scaled_LnIreland × − 0.403076) + (scaled_LnItaly × 0.419861) + (scaled_LnLuxembourg × − 0.784851) + (scaled_LnNetherland × − 0.791931) + (scaled_LnPortugal × 0.580261) + (scaled_LnSpain × − 0.0510254) + (scaled_AustriaS × − 0.227234) + (scaled_Belgium × 0.354248) + (scaled_Filand × − 0.0770264) + (scaled_France × − 0.137695) + (scaled_Germany × − 0.647217) + (scaled_Greece × 0.220337) + (scaled_Ireland × 0.806824) + (scaled_Italy × 0.861755) + (scaled_Luxembourg × 0.108521) + (scaled_Netherland × 0.57019) + (scaled_Portugal × 0.269592) + (scaled_Spain × − 0.153198));
y_1_13 = Logistic (− 0.984375+ (scaled_LnGreece × 0.303162) + (scaled_LnIreland × 0.72821) + (scaled_LnItaly × 0.995178) + (scaled_LnLuxembourg × − 0.206177) + (scaled_LnNetherland × 0.922668) + (scaled_LnPortugal × 0.991821) + (scaled_LnSpain × − 0.673279) + (scaled_AustriaS × 0.967346) + (scaled_Belgium × 0.291748) + (scaled_Filand × 0.0319214) + (scaled_France × − 0.685181) + (scaled_Germany × 0.0255127) + (scaled_Greece × − 0.992798) + (scaled_Ireland × − 0.655396) + (scaled_Italy × − 0.387146) + (scaled_Luxembourg × 0.583069) + (scaled_Netherland × 0.499329) + (scaled_Portugal × 0.815674) + (scaled_Spain × − 0.966736));
y_1_14 = Logistic (− 0.877014+ (scaled_LnGreece × − 0.206604) + (scaled_LnIreland × − 0.464783) + (scaled_LnItaly × 0.46106) + (scaled_LnLuxembourg × − 0.423889) + (scaled_LnNetherland × 0.513123) + (scaled_LnPortugal × − 0.0240479) + (scaled_LnSpain × 0.405701) + (scaled_AustriaS × 0.742554) + (scaled_Belgium × − 0.389343) + (scaled_Filand × 0.0999146) + (scaled_France × 0.162964) + (scaled_Germany × − 0.0535889) + (scaled_Greece × − 0.0177612) + (scaled_Ireland × 0.805298) + (scaled_Italy × − 0.72229) + (scaled_Luxembourg × 0.835571) + (scaled_Netherland × 0.837341) + (scaled_Portugal × − 0.996155) + (scaled_Spain × 0.39447));
y_1_15 = Logistic (0.436279+ (scaled_LnGreece × 0.336487) + (scaled_LnIreland × − 0.18396) + (scaled_LnItaly × − 0.481567) + (scaled_LnLuxembourg × 0.81488) + (scaled_LnNetherland × 0.232971) + (scaled_LnPortugal × − 0.499084) + (scaled_LnSpain × − 0.202393) + (scaled_AustriaS × − 0.395081) + (scaled_Belgium × 0.093811) + (scaled_Filand × − 0.11377) + (scaled_France × 0.758118) + (scaled_Germany × 0.315918) + (scaled_Greece × − 0.640564) + (scaled_Ireland × − 0.633789) + (scaled_Italy × 0.069458) + (scaled_Luxembourg × 0.381409) + (scaled_Netherland × − 0.598022) + (scaled_Portugal × − 0.194092) + (scaled_Spain × − 0.807495));
y_1_16 = Logistic (− 0.0708008+ (scaled_LnGreece × − 0.747742) + (scaled_LnIreland × − 0.629639) + (scaled_LnItaly × 0.256042) + (scaled_LnLuxembourg × 0.483887) + (scaled_LnNetherland × 0.332092) + (scaled_LnPortugal × 0.632446) + (scaled_LnSpain × 0.875122) + (scaled_AustriaS × − 0.320251) + (scaled_Belgium × 0.776611) + (scaled_Filand × − 0.371521) + (scaled_France × − 0.378784) + (scaled_Germany × 0.0708618) + (scaled_Greece × 0.218384) + (scaled_Ireland × − 0.0541382) + (scaled_Italy × − 0.724243) + (scaled_Luxembourg × − 0.31842) + (scaled_Netherland × 0.480103) + (scaled_Portugal × − 0.569641) + (scaled_Spain × − 0.638672));
y_1_17 = Logistic (− 0.864563+ (scaled_LnGreece × − 0.235046) + (scaled_LnIreland × − 0.661743) + (scaled_LnItaly × − 0.856201) + (scaled_LnLuxembourg × 0.517334) + (scaled_LnNetherland × − 0.0725098) + (scaled_LnPortugal × 0.400513) + (scaled_LnSpain × − 0.0170898) + (scaled_AustriaS × − 0.10553) + (scaled_Belgium × 0.194336) + (scaled_Filand × 0.158752) + (scaled_France × − 0.229797) + (scaled_Germany × − 0.868103) + (scaled_Greece × − 0.670898) + (scaled_Ireland × 0.28009) + (scaled_Italy × 0.630127) + (scaled_Luxembourg × − 0.521912) + (scaled_Netherland × − 0.280151) + (scaled_Portugal × 0.199097) + (scaled_Spain × − 0.536926));
y_1_18 = Logistic (− 0.488464+ (scaled_LnGreece × − 0.380432) + (scaled_LnIreland × 0.143799) + (scaled_LnItaly × 0.0730591) + (scaled_LnLuxembourg × − 0.670654) + (scaled_LnNetherland × 0.778809) + (scaled_LnPortugal × 0.723938) + (scaled_LnSpain × − 0.836853) + (scaled_AustriaS × 0.555908) + (scaled_Belgium × 0.64386) + (scaled_Filand × − 0.611023) + (scaled_France × − 0.296265) + (scaled_Germany × − 0.621887) + (scaled_Greece × 0.693237) + (scaled_Ireland × 0.150024) + (scaled_Italy × − 0.67749) + (scaled_Luxembourg × 0.929382) + (scaled_Netherland × 0.476868) + (scaled_Portugal × 0.506104) + (scaled_Spain × 0.0740356));
y_1_19 = Logistic (0.24939+ (scaled_LnGreece × 0.390625) + (scaled_LnIreland × − 0.582214) + (scaled_LnItaly × 0.29126) + (scaled_LnLuxembourg × − 0.850952) + (scaled_LnNetherland × − 0.887146) + (scaled_LnPortugal × − 0.305359) + (scaled_LnSpain × − 0.725037) + (scaled_AustriaS × − 0.678284) + (scaled_Belgium × − 0.475708) + (scaled_Filand × 0.76947) + (scaled_France × − 0.170288) + (scaled_Germany × 0.198181) + (scaled_Greece × − 0.364136) + (scaled_Ireland × − 0.877502) + (scaled_Italy × − 0.0227661) + (scaled_Luxembourg × 0.32782) + (scaled_Netherland × 0.956238) + (scaled_Portugal × − 0.244141) + (scaled_Spain × − 0.87439));
y_1_20 = Logistic (− 0.11438+ (scaled_LnGreece × − 0.758972) + (scaled_LnIreland × 0.629883) + (scaled_LnItaly × − 0.969727) + (scaled_LnLuxembourg × − 0.188904) + (scaled_LnNetherland × − 0.525391) + (scaled_LnPortugal × − 0.390015) + (scaled_LnSpain × − 0.604553) + (scaled_AustriaS × − 0.24408) + (scaled_Belgium × − 0.352051) + (scaled_Filand × − 0.320435) + (scaled_France × − 0.422119) + (scaled_Germany × − 0.445068) + (scaled_Greece × 0.65332) + (scaled_Ireland × − 0.868103) + (scaled_Italy × 0.788147) + (scaled_Luxembourg × 0.303955) + (scaled_Netherland × 0.020752) + (scaled_Portugal × − 0.0753784) + (scaled_Spain × 0.510254));
y_1_21 = Logistic (0.0829468+ (scaled_LnGreece × 0.481628) + (scaled_LnIreland × 0.522156) + (scaled_LnItaly × 0.11792) + (scaled_LnLuxembourg × 0.435425) + (scaled_LnNetherland × 0.398071) + (scaled_LnPortugal × 0.0758057) + (scaled_LnSpain × 0.411255) + (scaled_AustriaS × 0.102966) + (scaled_Belgium × − 0.477661) + (scaled_Filand × − 0.986023) + (scaled_France × 0.589783) + (scaled_Germany × 0.810669) + (scaled_Greece × − 0.999084) + (scaled_Ireland × − 0.223267) + (scaled_Italy × 0.693665) + (scaled_Luxembourg × − 0.6427) + (scaled_Netherland × 0.680847) + (scaled_Portugal × − 0.906067) + (scaled_Spain × 0.722168));
y_1_22 = Logistic (− 0.793152+ (scaled_LnGreece × 0.179504) + (scaled_LnIreland × 0.948425) + (scaled_LnItaly × − 0.282471) + (scaled_LnLuxembourg × − 0.314209) + (scaled_LnNetherland × − 0.554199) + (scaled_LnPortugal × 0.69751) + (scaled_LnSpain × − 0.2323) + (scaled_AustriaS × − 0.181458) + (scaled_Belgium × − 0.329834) + (scaled_Filand × − 0.261169) + (scaled_France × 0.754761) + (scaled_Germany × 0.762573) + (scaled_Greece × 0.40741) + (scaled_Ireland × 0.963318) + (scaled_Italy × 0.338928) + (scaled_Luxembourg × − 0.461731) + (scaled_Netherland × − 0.406433) + (scaled_Portugal × 0.571838) + (scaled_Spain × 0.891052));
y_1_23 = Logistic (0.585205+ (scaled_LnGreece × − 0.888123) + (scaled_LnIreland × − 0.907166) + (scaled_LnItaly × − 0.316101) + (scaled_LnLuxembourg × − 0.0828857) + (scaled_LnNetherland × − 0.626953) + (scaled_LnPortugal × − 0.985901) + (scaled_LnSpain × − 0.362427) + (scaled_AustriaS × − 0.592529) + (scaled_Belgium × − 0.664307) + (scaled_Filand × 0.359863) + (scaled_France × 0.293701) + (scaled_Germany × 0.175598) + (scaled_Greece × 0.373291) + (scaled_Ireland × 0.569336) + (scaled_Italy × − 0.344177) + (scaled_Luxembourg × 0.24646) + (scaled_Netherland × − 0.697937) + (scaled_Portugal × − 0.481323) + (scaled_Spain × 0.119873));
y_2_1 = Logistic (− 0.72052+ (y_1_1 × − 0.492554) + (y_1_2 × 0.645752) + (y_1_3 × − 0.715454) + (y_1_4 × 0.901245) + (y_1_5 × − 0.631287) + (y_1_6 × − 0.737366) + (y_1_7 × 0.945007) + (y_1_8 × − 0.953064) + (y_1_9 × 0.570618) + (y_1_10 × − 0.317993) + (y_1_11 × 0.787537) + (y_1_12 × 0.689636) + (y_1_13 × 0.713074) + (y_1_14 × 0.296265) + (y_1_15 × − 0.941833) + (y_1_16 × − 0.176819) + (y_1_17 × 0.620972) + (y_1_18 × − 0.786072) + (y_1_19 × 0.681763) + (y_1_20 × 0.919739) + (y_1_21 × 0.135986) + (y_1_22 × 0.133545) + (y_1_23 × − 0.107605));
y_2_2 = Logistic (0.595642+ (y_1_1 × 0.290039) + (y_1_2 × 0.288025) + (y_1_3 × − 0.92041) + (y_1_4 × 0.273376) + (y_1_5 × − 0.787659) + (y_1_6 × 0.157654) + (y_1_7 × − 0.934692) + (y_1_8 × − 0.979553) + (y_1_9 × 0.393311) + (y_1_10 × − 0.257446) + (y_1_11 × 0.743225) + (y_1_12 × − 0.760315) + (y_1_13 × 0.32428) + (y_1_14 × − 0.859558) + (y_1_15 × 0.106384) + (y_1_16 × − 0.906555) + (y_1_17 × − 0.906433) + (y_1_18 × − 0.639343) + (y_1_19 × − 0.696777) + (y_1_20 × − 0.393921) + (y_1_21 × 0.218811) + (y_1_22 × − 0.922363) + (y_1_23 × − 0.371948));
y_2_3 = Logistic (− 0.927429+ (y_1_1 × − 0.0574341) + (y_1_2 × 0.0614624) + (y_1_3 × 0.637695) + (y_1_4 × 0.749756) + (y_1_5 × − 0.02771) + (y_1_6 × − 0.614441) + (y_1_7 × − 0.367188) + (y_1_8 × − 0.758667) + (y_1_9 × 0.930359) + (y_1_10 × 0.623413) + (y_1_11 × − 0.647278) + (y_1_12 × 0.472717) + (y_1_13 × − 0.0109253) + (y_1_14 × − 0.588745) + (y_1_15 × 0.428772) + (y_1_16 × 0.929382) + (y_1_17 × − 0.447571) + (y_1_18 × − 0.043457) + (y_1_19 × 0.981995) + (y_1_20 × − 0.980713) + (y_1_21 × − 0.9151) + (y_1_22 × 0.799866) + (y_1_23 × − 0.413391));
y_2_4 = Logistic (0.317627+ (y_1_1 × − 0.569458) + (y_1_2 × 0.289063) + (y_1_3 × 0.534058) + (y_1_4 × 0.363586) + (y_1_5 × − 0.897827) + (y_1_6 × − 0.557739) + (y_1_7 × 0.0427246) + (y_1_8 × 0.514709) + (y_1_9 × − 0.128906) + (y_1_10 × − 0.293274) + (y_1_11 × 0.82074) + (y_1_12 × − 0.388855) + (y_1_13 × − 0.143005) + (y_1_14 × − 0.775635) + (y_1_15 × − 0.06073) + (y_1_16 × 0.947937) + (y_1_17 × 0.648132) + (y_1_18 × − 0.19043) + (y_1_19 × − 0.781921) + (y_1_20 × 0.277954) + (y_1_21 × 0.350403) + (y_1_22 × 0.0517578) + (y_1_23 × − 0.833008));
y_2_5 = Logistic (− 0.301453+ (y_1_1 × 0.236633) + (y_1_2 × − 0.437744) + (y_1_3 × − 0.358887) + (y_1_4 × − 0.740356) + (y_1_5 × − 0.228882) + (y_1_6 × 0.337708) + (y_1_7 × 0.728821) + (y_1_8 × − 0.898193) + (y_1_9 × 0.885559) + (y_1_10 × − 0.799622) + (y_1_11 × − 0.864258) + (y_1_12 × 0.0564575) + (y_1_13 × 0.0155029) + (y_1_14 × 0.270691) + (y_1_15 × − 0.779663) + (y_1_16 × − 0.11145) + (y_1_17 × 0.343262) + (y_1_18 × − 0.431213) + (y_1_19 × − 0.779785) + (y_1_20 × − 0.00793457) + (y_1_21 × 0.36792) + (y_1_22 × 0.555664) + (y_1_23 × 0.877991));
y_2_6 = Logistic (− 0.223267+ (y_1_1 × − 0.238892) + (y_1_2 × − 0.959167) + (y_1_3 × − 0.0244751) + (y_1_4 × 0.165283) + (y_1_5 × − 0.331665) + (y_1_6 × 0.781067) + (y_1_7 × 0.0608521) + (y_1_8 × 0.916992) + (y_1_9 × − 0.387207) + (y_1_10 × 0.681213) + (y_1_11 × 0.163696) + (y_1_12 × 0.187439) + (y_1_13 × 0.100098) + (y_1_14 × − 0.782227) + (y_1_15 × − 0.125305) + (y_1_16 × − 0.8573) + (y_1_17 × 0.81665) + (y_1_18 × 0.777954) + (y_1_19 × 0.693787) + (y_1_20 × 0.299988) + (y_1_21 × 0.161194) + (y_1_22 × − 0.685181) + (y_1_23 × 0.313293));
y_2_7 = Logistic (− 0.828735+ (y_1_1 × 0.927185) + (y_1_2 × 0.295837) + (y_1_3 × − 0.55542) + (y_1_4 × 0.577026) + (y_1_5 × − 0.584839) + (y_1_6 × − 0.720886) + (y_1_7 × 0.297119) + (y_1_8 × − 0.821777) + (y_1_9 × 0.435547) + (y_1_10 × 0.843689) + (y_1_11 × 0.433533) + (y_1_12 × 0.157593) + (y_1_13 × − 0.304626) + (y_1_14 × − 0.619202) + (y_1_15 × − 0.858093) + (y_1_16 × − 0.735229) + (y_1_17 × 0.102783) + (y_1_18 × 0.694519) + (y_1_19 × 0.425171) + (y_1_20 × − 0.75415) + (y_1_21 × 0.154724) + (y_1_22 × 0.0877686) + (y_1_23 × 0.992493));
y_2_8 = Logistic (0.22467+ (y_1_1 × − 0.880859) + (y_1_2 × 0.360229) + (y_1_3 × 0.343262) + (y_1_4 × 0.566956) + (y_1_5 × 0.50116) + (y_1_6 × 0.510864) + (y_1_7 × − 0.838745) + (y_1_8 × − 0.177368) + (y_1_9 × − 0.953247) + (y_1_10 × − 0.387939) + (y_1_11 × 0.616516) + (y_1_12 × 0.262512) + (y_1_13 × 0.0643311) + (y_1_14 × − 0.713745) + (y_1_15 × 0.197937) + (y_1_16 × − 0.649475) + (y_1_17 × 0.369751) + (y_1_18 × 0.461975) + (y_1_19 × − 0.00775146) + (y_1_20 × − 0.789612) + (y_1_21 × − 0.559143) + (y_1_22 × 0.159973) + (y_1_23 × − 0.425171));
y_2_9 = Logistic (− 0.0188599+ (y_1_1 × 0.202637) + (y_1_2 × − 0.0648193) + (y_1_3 × 0.189697) + (y_1_4 × − 0.829651) + (y_1_5 × − 0.306396) + (y_1_6 × 0.633545) + (y_1_7 × 0.568909) + (y_1_8 × 0.644897) + (y_1_9 × − 0.671875) + (y_1_10 × − 0.127808) + (y_1_11 × − 0.522461) + (y_1_12 × 0.893127) + (y_1_13 × − 0.881592) + (y_1_14 × 0.525208) + (y_1_15 × 0.559875) + (y_1_16 × − 0.81781) + (y_1_17 × − 0.337463) + (y_1_18 × 0.600464) + (y_1_19 × − 0.613159) + (y_1_20 × − 0.984741) + (y_1_21 × 0.803345) + (y_1_22 × 0.511658) + (y_1_23 × − 0.109375));
y_2_10 = Logistic (− 0.922119+ (y_1_1 × 0.0771484) + (y_1_2 × 0.0422363) + (y_1_3 × − 0.91217) + (y_1_4 × 0.584839) + (y_1_5 × 0.824341) + (y_1_6 × − 0.981567) + (y_1_7 × − 0.615479) + (y_1_8 × − 0.618164) + (y_1_9 × − 0.717834) + (y_1_10 × − 0.631836) + (y_1_11 × 0.357727) + (y_1_12 × 0.820923) + (y_1_13 × 0.611206) + (y_1_14 × 0.27417) + (y_1_15 × − 0.536133) + (y_1_16 × 0.518372) + (y_1_17 × − 0.99353) + (y_1_18 × − 0.97998) + (y_1_19 × 0.247681) + (y_1_20 × 0.65332) + (y_1_21 × 0.490112) + (y_1_22 × 0.860474) + (y_1_23 × − 0.0187378));
y_2_11 = Logistic (0.340637+ (y_1_1 × − 0.699036) + (y_1_2 × 0.161926) + (y_1_3 × − 0.479492) + (y_1_4 × − 0.583557) + (y_1_5 × − 0.574219) + (y_1_6 × 0.469055) + (y_1_7 × 0.636047) + (y_1_8 × − 0.287537) + (y_1_9 × 0.3479) + (y_1_10 × 0.0579224) + (y_1_11 × − 0.551086) + (y_1_12 × 0.602295) + (y_1_13 × − 0.506104) + (y_1_14 × − 0.229736) + (y_1_15 × − 0.9328) + (y_1_16 × 0.860046) + (y_1_17 × − 0.753723) + (y_1_18 × − 0.653076) + (y_1_19 × 0.605652) + (y_1_20 × − 0.869324) + (y_1_21 × 0.701843) + (y_1_22 × − 0.599609) + (y_1_23 × − 0.483154));
y_2_12 = Logistic (0.939392+ (y_1_1 × 0.186584) + (y_1_2 × − 0.846069) + (y_1_3 × 0.219788) + (y_1_4 × − 0.573792) + (y_1_5 × 0.671875) + (y_1_6 × − 0.891174) + (y_1_7 × − 0.0293579) + (y_1_8 × − 0.216125) + (y_1_9 × − 0.602722) + (y_1_10 × − 0.349304) + (y_1_11 × − 0.149048) + (y_1_12 × 0.64502) + (y_1_13 × 0.249023) + (y_1_14 × − 0.0342407) + (y_1_15 × 0.767517) + (y_1_16 × 0.891907) + (y_1_17 × 0.772644) + (y_1_18 × − 0.159912) + (y_1_19 × − 0.906189) + (y_1_20 × − 0.790283) + (y_1_21 × 0.49585) + (y_1_22 × 0.941772) + (y_1_23 × 0.0726929));
y_2_13 = Logistic (0.844421+ (y_1_1 × 0.12854) + (y_1_2 × 0.0515747) + (y_1_3 × − 0.299011) + (y_1_4 × 0.425964) + (y_1_5 × 0.445007) + (y_1_6 × − 0.835449) + (y_1_7 × − 0.399292) + (y_1_8 × 0.733582) + (y_1_9 × 0.334351) + (y_1_10 × 0.563904) + (y_1_11 × − 0.311035) + (y_1_12 × − 0.855225) + (y_1_13 × 0.467041) + (y_1_14 × − 0.116516) + (y_1_15 × − 0.872253) + (y_1_16 × 0.983704) + (y_1_17 × − 0.558167) + (y_1_18 × 0.613464) + (y_1_19 × 0.588013) + (y_1_20 × − 0.187683) + (y_1_21 × 0.847229) + (y_1_22 × 0.165039) + (y_1_23 × 0.306091));
y_2_14 = Logistic (0.438293+ (y_1_1 × 0.512573) + (y_1_2 × 0.264771) + (y_1_3 × 0.110718) + (y_1_4 × − 0.388) + (y_1_5 × − 0.0490723) + (y_1_6 × − 0.473694) + (y_1_7 × 0.663452) + (y_1_8 × − 0.256714) + (y_1_9 × − 0.213318) + (y_1_10 × 0.537048) + (y_1_11 × 0.455688) + (y_1_12 × 0.756348) + (y_1_13 × 0.988464) + (y_1_14 × − 0.0215454) + (y_1_15 × − 0.317383) + (y_1_16 × 0.3479) + (y_1_17 × 0.619202) + (y_1_18 × − 0.79834) + (y_1_19 × − 0.329468) + (y_1_20 × − 0.600525) + (y_1_21 × 0.453735) + (y_1_22 × 0.625427) + (y_1_23 × 0.933899));
y_2_15 = Logistic (− 0.509216+ (y_1_1 × 0.0562134) + (y_1_2 × − 0.00939941) + (y_1_3 × 0.950073) + (y_1_4 × 0.782776) + (y_1_5 × − 0.84552) + (y_1_6 × − 0.578552) + (y_1_7 × 0.50061) + (y_1_8 × 0.543945) + (y_1_9 × 0.808533) + (y_1_10 × − 0.881042) + (y_1_11 × − 0.988953) + (y_1_12 × − 0.542297) + (y_1_13 × 0.970642) + (y_1_14 × − 0.792114) + (y_1_15 × − 0.837158) + (y_1_16 × − 0.687439) + (y_1_17 × − 0.224121) + (y_1_18 × − 0.219971) + (y_1_19 × 0.189636) + (y_1_20 × 0.881409) + (y_1_21 × 0.484924) + (y_1_22 × − 0.683777) + (y_1_23 × 0.860779));
y_2_16 = Logistic (0.548584+ (y_1_1 × 0.220398) + (y_1_2 × 0.83667) + (y_1_3 × − 0.121948) + (y_1_4 × 0.156494) + (y_1_5 × − 0.106323) + (y_1_6 × − 0.376343) + (y_1_7 × 0.631531) + (y_1_8 × 0.946777) + (y_1_9 × − 0.813232) + (y_1_10 × 0.302979) + (y_1_11 × 0.265381) + (y_1_12 × 0.986816) + (y_1_13 × 0.370422) + (y_1_14 × 0.454041) + (y_1_15 × − 0.133667) + (y_1_16 × 0.541199) + (y_1_17 × − 0.922363) + (y_1_18 × 0.897461) + (y_1_19 × 0.813538) + (y_1_20 × 0.949463) + (y_1_21 × − 0.143066) + (y_1_22 × − 0.714355) + (y_1_23 × − 0.744141));
y_2_17 = Logistic (0.575928+ (y_1_1 × − 0.554016) + (y_1_2 × 0.963257) + (y_1_3 × − 0.0204468) + (y_1_4 × − 0.26709) + (y_1_5 × 0.545715) + (y_1_6 × − 0.549561) + (y_1_7 × − 0.977905) + (y_1_8 × − 0.496582) + (y_1_9 × − 0.690735) + (y_1_10 × 0.626038) + (y_1_11 × 0.0224609) + (y_1_12 × 0.143311) + (y_1_13 × 0.798889) + (y_1_14 × 0.932983) + (y_1_15 × − 0.237427) + (y_1_16 × − 0.0244751) + (y_1_17 × − 0.960938) + (y_1_18 × 0.31842) + (y_1_19 × − 0.660828) + (y_1_20 × − 0.10553) + (y_1_21 × − 0.207153) + (y_1_22 × − 0.845764) + (y_1_23 × − 0.346741));
y_2_18 = Logistic (0.351685+ (y_1_1 × 0.147461) + (y_1_2 × 0.467529) + (y_1_3 × − 0.229492) + (y_1_4 × − 0.940369) + (y_1_5 × 0.635986) + (y_1_6 × − 0.538574) + (y_1_7 × − 0.335632) + (y_1_8 × − 0.0107422) + (y_1_9 × 0.298401) + (y_1_10 × 0.264893) + (y_1_11 × 0.448059) + (y_1_12 × 0.35675) + (y_1_13 × 0.137878) + (y_1_14 × 0.62738) + (y_1_15 × − 0.403259) + (y_1_16 × 0.565613) + (y_1_17 × − 0.543213) + (y_1_18 × 0.103699) + (y_1_19 × − 0.939697) + (y_1_20 × − 0.0766602) + (y_1_21 × − 0.139099) + (y_1_22 × 0.291382) + (y_1_23 × − 0.19928));
y_3_1 = Logistic (− 0.275818+ (y_2_1 × 0.071228) + (y_2_2 × 0.911987) + (y_2_3 × − 0.0276489) + (y_2_4 × 0.300232) + (y_2_5 × − 0.980957) + (y_2_6 × 0.0669556) + (y_2_7 × − 0.624268) + (y_2_8 × 0.89563) + (y_2_9 × 0.586365) + (y_2_10 × − 0.00372314) + (y_2_11 × − 0.380066) + (y_2_12 × 0.289063) + (y_2_13 × − 0.640808) + (y_2_14 × 0.821594) + (y_2_15 × − 0.299744) + (y_2_16 × 0.414185) + (y_2_17 × − 0.219727) + (y_2_18 × − 0.514404));
y_3_2 = Logistic (0.763062+ (y_2_1 × 0.977295) + (y_2_2 × − 0.376404) + (y_2_3 × 0.487854) + (y_2_4 × − 0.0155029) + (y_2_5 × 0.729126) + (y_2_6 × − 0.71698) + (y_2_7 × 0.784058) + (y_2_8 × − 0.786194) + (y_2_9 × 0.863586) + (y_2_10 × 0.88501) + (y_2_11 × − 0.456055) + (y_2_12 × − 0.509583) + (y_2_13 × 0.602966) + (y_2_14 × − 0.755798) + (y_2_15 × − 0.120789) + (y_2_16 × − 0.1875) + (y_2_17 × 0.953857) + (y_2_18 × 0.337585));
y_3_3 = Logistic (− 0.104614+ (y_2_1 × − 0.132324) + (y_2_2 × 0.920166) + (y_2_3 × − 0.224915) + (y_2_4 × − 0.648438) + (y_2_5 × 0.662048) + (y_2_6 × 0.219543) + (y_2_7 × − 0.74469) + (y_2_8 × − 0.501343) + (y_2_9 × 0.289856) + (y_2_10 × − 0.367188) + (y_2_11 × 0.613342) + (y_2_12 × − 0.833984) + (y_2_13 × 0.457581) + (y_2_14 × 0.436218) + (y_2_15 × − 0.165955) + (y_2_16 × − 0.0410156) + (y_2_17 × 0.670898) + (y_2_18 × 0.148987));
y_3_4 = Logistic (0.383301+ (y_2_1 × − 0.748718) + (y_2_2 × − 0.799072) + (y_2_3 × 0.983826) + (y_2_4 × − 0.464478) + (y_2_5 × 0.791504) + (y_2_6 × − 0.546753) + (y_2_7 × − 0.310364) + (y_2_8 × 0.783447) + (y_2_9 × − 0.310181) + (y_2_10 × 0.617188) + (y_2_11 × − 0.734619) + (y_2_12 × 0.919739) + (y_2_13 × 0.49115) + (y_2_14 × − 0.819824) + (y_2_15 × 0.882751) + (y_2_16 × − 0.472046) + (y_2_17 × 0.149109) + (y_2_18 × 0.168945));
y_3_5 = Logistic (0.369446+ (y_2_1 × 0.798035) + (y_2_2 × 0.828308) + (y_2_3 × 0.0349731) + (y_2_4 × 0.372925) + (y_2_5 × 0.933472) + (y_2_6 × − 0.815918) + (y_2_7 × − 0.330383) + (y_2_8 × 0.39856) + (y_2_9 × 0.962463) + (y_2_10 × 0.0956421) + (y_2_11 × − 0.0100098) + (y_2_12 × 0.119385) + (y_2_13 × 0.210999) + (y_2_14 × − 0.253967) + (y_2_15 × 0.96405) + (y_2_16 × − 0.91803) + (y_2_17 × 0.688477) + (y_2_18 × − 0.841003));
y_3_6 = Logistic (− 0.251953+ (y_2_1 × − 0.63501) + (y_2_2 × 0.432495) + (y_2_3 × − 0.440735) + (y_2_4 × 0.0335083) + (y_2_5 × − 0.481201) + (y_2_6 × − 0.0757446) + (y_2_7 × 0.368958) + (y_2_8 × 0.316101) + (y_2_9 × 0.836731) + (y_2_10 × 0.570618) + (y_2_11 × − 0.663757) + (y_2_12 × 0.574341) + (y_2_13 × 0.945435) + (y_2_14 × 0.504517) + (y_2_15 × 0.0241089) + (y_2_16 × − 0.766785) + (y_2_17 × 0.227051) + (y_2_18 × 0.607666));
y_3_7 = Logistic (− 0.458313+ (y_2_1 × 0.702637) + (y_2_2 × − 0.262451) + (y_2_3 × 0.582397) + (y_2_4 × − 0.528381) + (y_2_5 × − 0.28479) + (y_2_6 × − 0.297302) + (y_2_7 × 0.228943) + (y_2_8 × 0.253662) + (y_2_9 × − 0.838501) + (y_2_10 × − 0.884033) + (y_2_11 × − 0.653503) + (y_2_12 × 0.989319) + (y_2_13 × 0.209045) + (y_2_14 × − 0.348877) + (y_2_15 × 0.383301) + (y_2_16 × 0.924805) + (y_2_17 × 0.346741) + (y_2_18 × 0.159119));
y_3_8 = Logistic (− 0.448853+ (y_2_1 × − 0.165588) + (y_2_2 × 0.431396) + (y_2_3 × − 0.108337) + (y_2_4 × 0.396667) + (y_2_5 × − 0.694458) + (y_2_6 × − 0.935913) + (y_2_7 × 0.0626221) + (y_2_8 × 0.0559692) + (y_2_9 × 0.674316) + (y_2_10 × − 0.672546) + (y_2_11 × − 0.0674438) + (y_2_12 × − 0.84259) + (y_2_13 × − 0.837952) + (y_2_14 × 0.711182) + (y_2_15 × 0.572449) + (y_2_16 × 0.867981) + (y_2_17 × − 0.872375) + (y_2_18 × − 0.442444));
y_3_9 = Logistic (0.815491+ (y_2_1 × − 0.301147) + (y_2_2 × 0.808655) + (y_2_3 × 0.396912) + (y_2_4 × − 0.676758) + (y_2_5 × − 0.862) + (y_2_6 × − 0.0883789) + (y_2_7 × − 0.0599365) + (y_2_8 × 0.798279) + (y_2_9 × 0.419067) + (y_2_10 × 0.891235) + (y_2_11 × 0.708252) + (y_2_12 × 0.844788) + (y_2_13 × 0.80835) + (y_2_14 × − 0.608826) + (y_2_15 × 0.443787) + (y_2_16 × − 0.782654) + (y_2_17 × − 0.902466) + (y_2_18 × − 0.131042));
y_3_10 = Logistic (− 0.247559+ (y_2_1 × 0.274963) + (y_2_2 × 0.0282593) + (y_2_3 × − 0.541138) + (y_2_4 × − 0.718079) + (y_2_5 × − 0.0913696) + (y_2_6 × − 0.580505) + (y_2_7 × 0.678223) + (y_2_8 × 0.636475) + (y_2_9 × − 0.766907) + (y_2_10 × 0.0043335) + (y_2_11 × − 0.919495) + (y_2_12 × − 0.537415) + (y_2_13 × − 0.856384) + (y_2_14 × − 0.358704) + (y_2_15 × 0.458679) + (y_2_16 × − 0.722717) + (y_2_17 × − 0.597229) + (y_2_18 × − 0.897888));
y_3_11 = Logistic (− 0.0736694+ (y_2_1 × − 0.681152) + (y_2_2 × − 0.901733) + (y_2_3 × 0.348816) + (y_2_4 × 0.19696) + (y_2_5 × 0.754639) + (y_2_6 × − 0.92865) + (y_2_7 × 0.264221) + (y_2_8 × 0.919434) + (y_2_9 × − 0.737122) + (y_2_10 × − 0.726746) + (y_2_11 × − 0.783386) + (y_2_12 × − 0.826782) + (y_2_13 × − 0.186646) + (y_2_14 × − 0.247009) + (y_2_15 × − 0.815063) + (y_2_16 × − 0.356628) + (y_2_17 × − 0.515564) + (y_2_18 × 0.873779));
y_3_12 = Logistic (− 0.0736694+ (y_2_1 × − 0.716553) + (y_2_2 × − 0.625122) + (y_2_3 × 0.978271) + (y_2_4 × 0.0186157) + (y_2_5 × − 0.104248) + (y_2_6 × 0.620361) + (y_2_7 × 0.326965) + (y_2_8 × − 0.278931) + (y_2_9 × 0.531311) + (y_2_10 × 0.520142) + (y_2_11 × 0.102722) + (y_2_12 × − 0.715149) + (y_2_13 × − 0.78241) + (y_2_14 × 0.176636) + (y_2_15 × 0.444702) + (y_2_16 × 0.953796) + (y_2_17 × 0.239258) + (y_2_18 × 0.685791));
y_3_13 = Logistic (0.934753+ (y_2_1 × − 0.785828) + (y_2_2 × 0.69574) + (y_2_3 × 0.0380249) + (y_2_4 × 0.688293) + (y_2_5 × − 0.408203) + (y_2_6 × 0.661194) + (y_2_7 × 0.085022) + (y_2_8 × − 0.997498) + (y_2_9 × 0.428223) + (y_2_10 × − 0.618774) + (y_2_11 × 0.537964) + (y_2_12 × 0.902039) + (y_2_13 × − 0.338684) + (y_2_14 × 0.765625) + (y_2_15 × − 0.503296) + (y_2_16 × − 0.399597) + (y_2_17 × 0.112549) + (y_2_18 × − 0.0354614));
y_3_14 = Logistic (0.164307+ (y_2_1 × − 0.33197) + (y_2_2 × − 0.494873) + (y_2_3 × − 0.0256958) + (y_2_4 × − 0.84436) + (y_2_5 × − 0.745972) + (y_2_6 × 0.803955) + (y_2_7 × − 0.698669) + (y_2_8 × − 0.679932) + (y_2_9 × 0.875061) + (y_2_10 × − 0.0542603) + (y_2_11 × 0.818787) + (y_2_12 × − 0.931396) + (y_2_13 × − 0.659668) + (y_2_14 × − 0.422241) + (y_2_15 × 0.358826) + (y_2_16 × 0.58136) + (y_2_17 × 0.0789185) + (y_2_18 × 0.503479));
y_3_15 = Logistic (0.870728+ (y_2_1 × − 0.761108) + (y_2_2 × − 0.276489) + (y_2_3 × − 0.956421) + (y_2_4 × − 0.778564) + (y_2_5 × − 0.106262) + (y_2_6 × 0.398743) + (y_2_7 × − 0.101013) + (y_2_8 × − 0.390564) + (y_2_9 × − 0.925964) + (y_2_10 × 0.204041) + (y_2_11 × − 0.691528) + (y_2_12 × 0.315247) + (y_2_13 × − 0.0765991) + (y_2_14 × − 0.552734) + (y_2_15 × 0.130493) + (y_2_16 × 0.416809) + (y_2_17 × − 0.752319) + (y_2_18 × 0.590942));
y_3_16 = Logistic (− 0.911438+ (y_2_1 × − 0.662292) + (y_2_2 × 0.915161) + (y_2_3 × − 0.712585) + (y_2_4 × 0.184265) + (y_2_5 × 0.471436) + (y_2_6 × 0.62085) + (y_2_7 × 0.306763) + (y_2_8 × 0.345337) + (y_2_9 × − 0.556458) + (y_2_10 × − 0.00158691) + (y_2_11 × − 0.687988) + (y_2_12 × − 0.142395) + (y_2_13 × − 0.501221) + (y_2_14 × − 0.47052) + (y_2_15 × − 0.78949) + (y_2_16 × − 0.0588379) + (y_2_17 × 0.149475) + (y_2_18 × 0.158569));
y_4_1 = Logistic (0.827271+ (y_3_1 × − 0.143677) + (y_3_2 × 0.991211) + (y_3_3 × − 0.834656) + (y_3_4 × − 0.774048) + (y_3_5 × 0.336975) + (y_3_6 × 0.999512) + (y_3_7 × 0.402954) + (y_3_8 × 0.885681) + (y_3_9 × − 0.240112) + (y_3_10 × − 0.0336304) + (y_3_11 × 0.471924) + (y_3_12 × − 0.256653) + (y_3_13 × 0.0755005) + (y_3_14 × 0.107605) + (y_3_15 × − 0.477722) + (y_3_16 × − 0.0449219));
y_4_2 = Logistic (0.189453+ (y_3_1 × − 0.00384521) + (y_3_2 × 0.744202) + (y_3_3 × 0.217163) + (y_3_4 × 0.653748) + (y_3_5 × − 0.24292) + (y_3_6 × − 0.730713) + (y_3_7 × − 0.918518) + (y_3_8 × − 0.847595) + (y_3_9 × − 0.243774) + (y_3_10 × 0.478333) + (y_3_11 × 0.760925) + (y_3_12 × − 0.583008) + (y_3_13 × − 0.614502) + (y_3_14 × − 0.674683) + (y_3_15 × 0.00390625) + (y_3_16 × − 0.628479));
y_4_3 = Logistic (0.659485+ (y_3_1 × − 0.633911) + (y_3_2 × − 0.832825) + (y_3_3 × − 0.821899) + (y_3_4 × − 0.402588) + (y_3_5 × − 0.0999146) + (y_3_6 × 0.558899) + (y_3_7 × 0.135071) + (y_3_8 × 0.990051) + (y_3_9 × − 0.0491333) + (y_3_10 × 0.661987) + (y_3_11 × 0.235107) + (y_3_12 × − 0.0653687) + (y_3_13 × − 0.03479) + (y_3_14 × 0.328735) + (y_3_15 × − 0.655823) + (y_3_16 × 0.564514));
y_4_4 = Logistic (− 0.788513+ (y_3_1 × − 0.651184) + (y_3_2 × 0.924683) + (y_3_3 × − 0.38324) + (y_3_4 × 0.663513) + (y_3_5 × − 0.991028) + (y_3_6 × − 0.0481567) + (y_3_7 × 0.87561) + (y_3_8 × 0.775696) + (y_3_9 × 0.27533) + (y_3_10 × − 0.464539) + (y_3_11 × − 0.597473) + (y_3_12 × − 0.915527) + (y_3_13 × 0.512634) + (y_3_14 × 0.836243) + (y_3_15 × 0.950684) + (y_3_16 × − 0.376099));
y_4_5 = Logistic (− 0.226196+ (y_3_1 × 0.737061) + (y_3_2 × 0.450867) + (y_3_3 × − 0.206787) + (y_3_4 × 0.384216) + (y_3_5 × − 0.450684) + (y_3_6 × − 0.13623) + (y_3_7 × 0.618835) + (y_3_8 × 0.874573) + (y_3_9 × 0.696106) + (y_3_10 × 0.0114136) + (y_3_11 × − 0.912354) + (y_3_12 × − 0.294556) + (y_3_13 × 0.573486) + (y_3_14 × 0.0231323) + (y_3_15 × − 0.140259) + (y_3_16 × − 0.247009));
y_4_6 = Logistic (0.118164+ (y_3_1 × 0.983826) + (y_3_2 × 0.199463) + (y_3_3 × − 0.0909424) + (y_3_4 × − 0.532349) + (y_3_5 × − 0.540527) + (y_3_6 × 0.292297) + (y_3_7 × − 0.0656128) + (y_3_8 × − 0.109741) + (y_3_9 × 0.465088) + (y_3_10 × − 0.852905) + (y_3_11 × − 0.318481) + (y_3_12 × − 0.0628052) + (y_3_13 × − 0.208862) + (y_3_14 × 0.77179) + (y_3_15 × − 0.0131226) + (y_3_16 × − 0.481873));
y_4_7 = Logistic (− 0.0684814+ (y_3_1 × − 0.547852) + (y_3_2 × 0.893677) + (y_3_3 × − 0.643005) + (y_3_4 × − 0.952271) + (y_3_5 × − 0.599609) + (y_3_6 × − 0.932983) + (y_3_7 × − 0.104187) + (y_3_8 × 0.914612) + (y_3_9 × − 0.63092) + (y_3_10 × 0.394104) + (y_3_11 × 0.391418) + (y_3_12 × − 0.529724) + (y_3_13 × − 0.843323) + (y_3_14 × − 0.0783691) + (y_3_15 × − 0.62384) + (y_3_16 × − 0.900879));
y_4_8 = Logistic (0.130859+ (y_3_1 × 0.665344) + (y_3_2 × − 0.223816) + (y_3_3 × − 0.0285034) + (y_3_4 × 0.93396) + (y_3_5 × 0.00567627) + (y_3_6 × 0.0997314) + (y_3_7 × − 0.669434) + (y_3_8 × − 0.222168) + (y_3_9 × 0.980225) + (y_3_10 × − 0.151245) + (y_3_11 × 0.49115) + (y_3_12 × − 0.204041) + (y_3_13 × 0.258728) + (y_3_14 × 0.519104) + (y_3_15 × − 0.549072) + (y_3_16 × 0.539001));
y_4_9 = Logistic (− 0.921387+ (y_3_1 × − 0.443237) + (y_3_2 × 0.859253) + (y_3_3 × − 0.721863) + (y_3_4 × − 0.138123) + (y_3_5 × − 0.868408) + (y_3_6 × − 0.366028) + (y_3_7 × 0.0700073) + (y_3_8 × − 0.70166) + (y_3_9 × − 0.0941772) + (y_3_10 × 0.501831) + (y_3_11 × 0.57074) + (y_3_12 × 0.456238) + (y_3_13 × 0.53363) + (y_3_14 × 0.067749) + (y_3_15 × 0.263062) + (y_3_16 × − 0.00592041));
y_4_10 = Logistic (− 0.793457+ (y_3_1 × 0.720032) + (y_3_2 × − 0.825256) + (y_3_3 × − 0.25592) + (y_3_4 × − 0.257446) + (y_3_5 × − 0.893005) + (y_3_6 × 0.495667) + (y_3_7 × 0.199341) + (y_3_8 × − 0.467285) + (y_3_9 × − 0.961853) + (y_3_10 × 0.488586) + (y_3_11 × − 0.213135) + (y_3_12 × − 0.706055) + (y_3_13 × 0.515381) + (y_3_14 × 0.778015) + (y_3_15 × − 0.256348) + (y_3_16 × − 0.0160522));
y_4_11 = Logistic (0.0838623+ (y_3_1 × − 0.341125) + (y_3_2 × − 0.976074) + (y_3_3 × 0.027832) + (y_3_4 × − 0.0869141) + (y_3_5 × − 0.0164795) + (y_3_6 × 0.342468) + (y_3_7 × − 0.692139) + (y_3_8 × 0.026062) + (y_3_9 × 0.804871) + (y_3_10 × − 0.661011) + (y_3_11 × − 0.887634) + (y_3_12 × 0.702759) + (y_3_13 × − 0.914307) + (y_3_14 × − 0.182556) + (y_3_15 × 0.952515) + (y_3_16 × − 0.358582));
y_4_12 = Logistic (0.144348+ (y_3_1 × 0.0370483) + (y_3_2 × 0.227722) + (y_3_3 × 0.229248) + (y_3_4 × − 0.613831) + (y_3_5 × 0.7854) + (y_3_6 × − 0.0857544) + (y_3_7 × − 0.261475) + (y_3_8 × 0.0615845) + (y_3_9 × 0.418579) + (y_3_10 × 0.227356) + (y_3_11 × 0.586731) + (y_3_12 × − 0.122925) + (y_3_13 × 0.723816) + (y_3_14 × − 0.439392) + (y_3_15 × − 0.671509) + (y_3_16 × 0.962769));
y_4_13 = Logistic (− 0.54126+ (y_3_1 × 0.928772) + (y_3_2 × 0.376465) + (y_3_3 × − 0.860291) + (y_3_4 × − 0.899658) + (y_3_5 × 0.75946) + (y_3_6 × − 0.717163) + (y_3_7 × − 0.161621) + (y_3_8 × 0.910889) + (y_3_9 × − 0.872742) + (y_3_10 × 0.222595) + (y_3_11 × 0.565674) + (y_3_12 × − 0.937988) + (y_3_13 × 0.756775) + (y_3_14 × − 0.160889) + (y_3_15 × − 0.060791) + (y_3_16 × − 0.942688));
y_4_14 = Logistic (− 0.69165+ (y_3_1 × 0.0681152) + (y_3_2 × − 0.919861) + (y_3_3 × − 0.508118) + (y_3_4 × 0.169922) + (y_3_5 × 0.625305) + (y_3_6 × 0.102051) + (y_3_7 × − 0.791687) + (y_3_8 × 0.0837402) + (y_3_9 × − 0.536194) + (y_3_10 × 0.538147) + (y_3_11 × 0.971375) + (y_3_12 × − 0.196411) + (y_3_13 × 0.60907) + (y_3_14 × 0.19165) + (y_3_15 × 0.731262) + (y_3_16 × 0.397827));
y_4_15 = Logistic (0.540405+ (y_3_1 × − 0.900574) + (y_3_2 × 0.21814) + (y_3_3 × − 0.790039) + (y_3_4 × 0.13501) + (y_3_5 × − 0.174866) + (y_3_6 × − 0.142151) + (y_3_7 × − 0.046875) + (y_3_8 × − 0.421753) + (y_3_9 × 0.496521) + (y_3_10 × 0.781921) + (y_3_11 × 0.54248) + (y_3_12 × − 0.801453) + (y_3_13 × − 0.946899) + (y_3_14 × 0.113831) + (y_3_15 × − 0.219788) + (y_3_16 × − 0.929688));
y_5_1 = Logistic (− 0.793945+ (y_4_1 × − 0.976013) + (y_4_2 × 0.466248) + (y_4_3 × 0.162659) + (y_4_4 × − 0.102661) + (y_4_5 × 0.541016) + (y_4_6 × − 0.891174) + (y_4_7 × 0.782959) + (y_4_8 × 0.00610352) + (y_4_9 × − 0.533813) + (y_4_10 × − 0.901306) + (y_4_11 × 0.958923) + (y_4_12 × − 0.282959) + (y_4_13 × − 0.410645) + (y_4_14 × − 0.956421) + (y_4_15 × − 0.0650635));
y_5_2 = Logistic (0.0010376+ (y_4_1 × − 0.904785) + (y_4_2 × 0.321533) + (y_4_3 × 0.891235) + (y_4_4 × 0.325806) + (y_4_5 × − 0.078064) + (y_4_6 × 0.441101) + (y_4_7 × 0.940308) + (y_4_8 × 0.334167) + (y_4_9 × − 0.706604) + (y_4_10 × 0.54718) + (y_4_11 × 0.730286) + (y_4_12 × 0.33075) + (y_4_13 × 0.1297) + (y_4_14 × 0.782593) + (y_4_15 × 0.122925));
y_5_3 = Logistic (0.231567+ (y_4_1 × 0.860352) + (y_4_2 × − 0.593201) + (y_4_3 × − 0.611328) + (y_4_4 × − 0.112793) + (y_4_5 × 0.136353) + (y_4_6 × − 0.0769653) + (y_4_7 × 0.654846) + (y_4_8 × 0.101135) + (y_4_9 × 0.0858765) + (y_4_10 × 0.413757) + (y_4_11 × − 0.174927) + (y_4_12 × − 0.317688) + (y_4_13 × − 0.992004) + (y_4_14 × − 0.173828) + (y_4_15 × − 0.476013));
y_5_4 = Logistic (0.922974+ (y_4_1 × − 0.640381) + (y_4_2 × − 0.0915527) + (y_4_3 × 0.566284) + (y_4_4 × − 0.735413) + (y_4_5 × − 0.600769) + (y_4_6 × 0.571594) + (y_4_7 × 0.531738) + (y_4_8 × 0.601318) + (y_4_9 × 0.39679) + (y_4_10 × 0.434082) + (y_4_11 × 0.741638) + (y_4_12 × − 0.510559) + (y_4_13 × 0.448669) + (y_4_14 × 0.321411) + (y_4_15 × 0.377014));
y_5_5 = Logistic (0.0111084+ (y_4_1 × 0.414124) + (y_4_2 × − 0.985779) + (y_4_3 × 0.109985) + (y_4_4 × 0.0437622) + (y_4_5 × − 0.148315) + (y_4_6 × 0.782593) + (y_4_7 × 0.378052) + (y_4_8 × − 0.929321) + (y_4_9 × − 0.374634) + (y_4_10 × − 0.144592) + (y_4_11 × − 0.893005) + (y_4_12 × − 0.344849) + (y_4_13 × − 0.281921) + (y_4_14 × − 0.441589) + (y_4_15 × − 0.503967));
y_5_6 = Logistic (0.325195+ (y_4_1 × − 0.33075) + (y_4_2 × − 0.895508) + (y_4_3 × 0.194946) + (y_4_4 × − 0.0471802) + (y_4_5 × 0.93811) + (y_4_6 × 0.165649) + (y_4_7 × 0.0527954) + (y_4_8 × 0.321899) + (y_4_9 × 0.803772) + (y_4_10 × − 0.752502) + (y_4_11 × − 0.0222778) + (y_4_12 × − 0.585388) + (y_4_13 × − 0.213501) + (y_4_14 × 0.926941) + (y_4_15 × 0.825928));
y_5_7 = Logistic (0.451111+ (y_4_1 × − 0.0983276) + (y_4_2 × − 0.843689) + (y_4_3 × 0.547241) + (y_4_4 × 0.107727) + (y_4_5 × − 0.368164) + (y_4_6 × − 0.772766) + (y_4_7 × 0.585388) + (y_4_8 × − 0.864563) + (y_4_9 × − 0.561096) + (y_4_10 × 0.186523) + (y_4_11 × − 0.585999) + (y_4_12 × 0.987244) + (y_4_13 × 0.499084) + (y_4_14 × 0.271729) + (y_4_15 × 0.0205078));
y_5_8 = Logistic (0.457642+ (y_4_1 × 0.600586) + (y_4_2 × − 0.697571) + (y_4_3 × − 0.891907) + (y_4_4 × − 0.754578) + (y_4_5 × − 0.69397) + (y_4_6 × − 0.70105) + (y_4_7 × − 0.982178) + (y_4_8 × − 0.896729) + (y_4_9 × − 0.883789) + (y_4_10 × − 0.715759) + (y_4_11 × 0.11554) + (y_4_12 × 0.747131) + (y_4_13 × − 0.919922) + (y_4_14 × − 0.415283) + (y_4_15 × 0.000793457));
y_5_9 = Logistic (− 0.481079+ (y_4_1 × 0.641357) + (y_4_2 × − 0.983093) + (y_4_3 × 0.107117) + (y_4_4 × − 0.139282) + (y_4_5 × − 0.497192) + (y_4_6 × 0.94104) + (y_4_7 × − 0.686829) + (y_4_8 × − 0.204468) + (y_4_9 × − 0.425293) + (y_4_10 × − 0.508972) + (y_4_11 × 0.182678) + (y_4_12 × − 0.820801) + (y_4_13 × 0.794739) + (y_4_14 × − 0.325012) + (y_4_15 × 0.2276));
y_5_10 = Logistic (− 0.607422+ (y_4_1 × 0.867554) + (y_4_2 × − 0.579529) + (y_4_3 × − 0.755249) + (y_4_4 × − 0.380981) + (y_4_5 × − 0.933044) + (y_4_6 × − 0.326111) + (y_4_7 × 0.0116577) + (y_4_8 × 0.490417) + (y_4_9 × − 0.325012) + (y_4_10 × − 0.0314331) + (y_4_11 × − 0.583984) + (y_4_12 × − 0.521423) + (y_4_13 × 0.906982) + (y_4_14 × 0.280518) + (y_4_15 × 0.663391));
y_5_11 = Logistic (0.699402+ (y_4_1 × 0.56604) + (y_4_2 × − 0.980225) + (y_4_3 × 0.226929) + (y_4_4 × 0.738953) + (y_4_5 × 0.28595) + (y_4_6 × 0.945801) + (y_4_7 × 0.414063) + (y_4_8 × 0.614258) + (y_4_9 × 0.353699) + (y_4_10 × 0.649597) + (y_4_11 × 0.616943) + (y_4_12 × 0.957397) + (y_4_13 × − 0.214233) + (y_4_14 × − 0.980042) + (y_4_15 × − 0.646057));
y_5_12 = Logistic (0.693359+ (y_4_1 × − 0.340698) + (y_4_2 × − 0.429749) + (y_4_3 × 0.109131) + (y_4_4 × 0.418396) + (y_4_5 × 0.489502) + (y_4_6 × − 0.0496826) + (y_4_7 × − 0.467285) + (y_4_8 × 0.929443) + (y_4_9 × − 0.806885) + (y_4_10 × − 0.616943) + (y_4_11 × − 0.158264) + (y_4_12 × 0.465149) + (y_4_13 × 0.341492) + (y_4_14 × − 0.762451) + (y_4_15 × 0.680481));
y_5_13 = Logistic (− 0.126404+ (y_4_1 × − 0.159729) + (y_4_2 × − 0.750488) + (y_4_3 × − 0.707397) + (y_4_4 × 0.541138) + (y_4_5 × − 0.909546) + (y_4_6 × 0.271729) + (y_4_7 × 0.098877) + (y_4_8 × − 0.0704346) + (y_4_9 × − 0.773132) + (y_4_10 × − 0.209961) + (y_4_11 × − 0.734375) + (y_4_12 × 0.752747) + (y_4_13 × − 0.67804) + (y_4_14 × − 0.795105) + (y_4_15 × − 0.263184));
y_5_14 = Logistic (− 0.86908+ (y_4_1 × − 0.257568) + (y_4_2 × − 0.50946) + (y_4_3 × 0.243408) + (y_4_4 × − 0.882568) + (y_4_5 × 0.725403) + (y_4_6 × − 0.672729) + (y_4_7 × − 0.104065) + (y_4_8 × 0.113098) + (y_4_9 × − 0.445374) + (y_4_10 × 0.364014) + (y_4_11 × − 0.705627) + (y_4_12 × − 0.854614) + (y_4_13 × 0.512817) + (y_4_14 × 0.109192) + (y_4_15 × − 0.155762));
y_6_1 = Logistic (− 0.665161+ (y_5_1 × − 0.887817) + (y_5_2 × 0.46637) + (y_5_3 × − 0.162842) + (y_5_4 × 0.442871) + (y_5_5 × 0.416016) + (y_5_6 × 0.292969) + (y_5_7 × 0.771973) + (y_5_8 × − 0.492065) + (y_5_9 × 0.272644) + (y_5_10 × − 0.412537) + (y_5_11 × 0.236755) + (y_5_12 × − 0.755432) + (y_5_13 × 0.142273) + (y_5_14 × 0.321716));
y_6_2 = Logistic (0.994751+ (y_5_1 × 0.258362) + (y_5_2 × 0.745178) + (y_5_3 × 0.229309) + (y_5_4 × 0.438416) + (y_5_5 × 0.341675) + (y_5_6 × 0.496033) + (y_5_7 × − 0.927856) + (y_5_8 × − 0.66925) + (y_5_9 × 0.280518) + (y_5_10 × 0.194702) + (y_5_11 × − 0.659363) + (y_5_12 × 0.701355) + (y_5_13 × 0.831726) + (y_5_14 × 0.977966));
y_6_3 = Logistic (− 0.292419+ (y_5_1 × 0.402588) + (y_5_2 × − 0.888123) + (y_5_3 × − 0.68512) + (y_5_4 × 0.872498) + (y_5_5 × − 0.777649) + (y_5_6 × − 0.462646) + (y_5_7 × 0.200562) + (y_5_8 × − 0.720032) + (y_5_9 × 0.154175) + (y_5_10 × 0.236328) + (y_5_11 × − 0.250793) + (y_5_12 × − 0.689331) + (y_5_13 × − 0.807556) + (y_5_14 × 0.250244));
y_6_4 = Logistic (− 0.669434+ (y_5_1 × 0.763306) + (y_5_2 × − 0.223206) + (y_5_3 × − 0.758789) + (y_5_4 × − 0.156006) + (y_5_5 × 0.515442) + (y_5_6 × − 0.34137) + (y_5_7 × − 0.0335083) + (y_5_8 × − 0.799316) + (y_5_9 × − 0.0855713) + (y_5_10 × 0.855469) + (y_5_11 × 0.792114) + (y_5_12 × − 0.317627) + (y_5_13 × 0.972534) + (y_5_14 × 0.0348511));
y_6_5 = Logistic (− 0.339294+ (y_5_1 × 0.603333) + (y_5_2 × 0.970276) + (y_5_3 × 0.635559) + (y_5_4 × − 0.608704) + (y_5_5 × 0.719727) + (y_5_6 × 0.395752) + (y_5_7 × 0.0392456) + (y_5_8 × − 0.871704) + (y_5_9 × 0.700012) + (y_5_10 × − 0.224304) + (y_5_11 × − 0.746704) + (y_5_12 × − 0.525146) + (y_5_13 × − 0.458374) + (y_5_14 × 0.0641479));
y_6_6 = Logistic (0.622559+ (y_5_1 × 0.576965) + (y_5_2 × − 0.94989) + (y_5_3 × − 0.162842) + (y_5_4 × − 0.867249) + (y_5_5 × 0.447083) + (y_5_6 × − 0.611816) + (y_5_7 × − 0.549561) + (y_5_8 × 0.992554) + (y_5_9 × − 0.977478) + (y_5_10 × 0.435181) + (y_5_11 × 0.274475) + (y_5_12 × − 0.229858) + (y_5_13 × − 0.493164) + (y_5_14 × 0.831726));
y_6_7 = Logistic (0.230713+ (y_5_1 × 0.634155) + (y_5_2 × 0.749695) + (y_5_3 × 0.271118) + (y_5_4 × − 0.206604) + (y_5_5 × 0.895874) + (y_5_6 × − 0.205933) + (y_5_7 × − 0.159058) + (y_5_8 × 0.400574) + (y_5_9 × 0.846497) + (y_5_10 × − 0.542542) + (y_5_11 × − 0.191284) + (y_5_12 × − 0.888245) + (y_5_13 × 0.404846) + (y_5_14 × − 0.898193));
y_6_8 = Logistic (− 0.126465+ (y_5_1 × − 0.265869) + (y_5_2 × − 0.688782) + (y_5_3 × 0.227112) + (y_5_4 × − 0.670349) + (y_5_5 × − 0.90448) + (y_5_6 × − 0.294617) + (y_5_7 × − 0.230164) + (y_5_8 × 0.0459595) + (y_5_9 × 0.354919) + (y_5_10 × − 0.621948) + (y_5_11 × − 0.628052) + (y_5_12 × 0.461243) + (y_5_13 × 0.198181) + (y_5_14 × 0.319458));
y_6_9 = Logistic (0.698181+ (y_5_1 × − 0.0806885) + (y_5_2 × 0.348572) + (y_5_3 × − 0.986145) + (y_5_4 × − 0.760193) + (y_5_5 × 0.653503) + (y_5_6 × 0.86731) + (y_5_7 × − 0.327148) + (y_5_8 × − 0.519348) + (y_5_9 × 0.6875) + (y_5_10 × 0.265137) + (y_5_11 × − 0.328857) + (y_5_12 × 0.836426) + (y_5_13 × 0.791321) + (y_5_14 × 0.772217));
y_6_10 = Logistic (0.499146+ (y_5_1 × − 0.301819) + (y_5_2 × 0.0986938) + (y_5_3 × − 0.990662) + (y_5_4 × − 0.00140381) + (y_5_5 × 0.694275) + (y_5_6 × 0.135376) + (y_5_7 × 0.67218) + (y_5_8 × − 0.85144) + (y_5_9 × 0.457764) + (y_5_10 × 0.317871) + (y_5_11 × 0.365845) + (y_5_12 × − 0.636475) + (y_5_13 × − 0.133728) + (y_5_14 × 0.108154));
y_6_11 = Logistic (0.669128+ (y_5_1 × 0.420288) + (y_5_2 × 0.506409) + (y_5_3 × − 0.226746) + (y_5_4 × 0.618652) + (y_5_5 × − 0.520325) + (y_5_6 × − 0.299927) + (y_5_7 × 0.643127) + (y_5_8 × − 0.0428467) + (y_5_9 × − 0.282227) + (y_5_10 × − 0.0678711) + (y_5_11 × − 0.0130615) + (y_5_12 × − 0.830627) + (y_5_13 × 0.26001) + (y_5_14 × 0.038208));
y_6_12 = Logistic (− 0.751648+ (y_5_1 × − 0.338318) + (y_5_2 × 0.591919) + (y_5_3 × − 0.625) + (y_5_4 × 0.377563) + (y_5_5 × 0.0947876) + (y_5_6 × − 0.572449) + (y_5_7 × 0.998291) + (y_5_8 × − 0.488525) + (y_5_9 × − 0.502441) + (y_5_10 × 0.355835) + (y_5_11 × 0.150696) + (y_5_12 × 0.199158) + (y_5_13 × − 0.870972) + (y_5_14 × − 0.00842285));
y_6_13 = Logistic (− 0.179749+ (y_5_1 × 0.813354) + (y_5_2 × 0.0214233) + (y_5_3 × 0.673035) + (y_5_4 × − 0.997131) + (y_5_5 × 0.82251) + (y_5_6 × 0.946899) + (y_5_7 × 0.79126) + (y_5_8 × 0.702393) + (y_5_9 × − 0.653442) + (y_5_10 × − 0.848633) + (y_5_11 × 0.914551) + (y_5_12 × − 0.076355) + (y_5_13 × − 0.492859) + (y_5_14 × − 0.596436));
y_7_1 = Logistic (0.757751+ (y_6_1 × − 0.55603) + (y_6_2 × 0.749084) + (y_6_3 × 0.722046) + (y_6_4 × 0.887146) + (y_6_5 × − 0.106934) + (y_6_6 × 0.112671) + (y_6_7 × − 0.481812) + (y_6_8 × 0.779602) + (y_6_9 × 0.54895) + (y_6_10 × − 0.381775) + (y_6_11 × − 0.835876) + (y_6_12 × − 0.391846) + (y_6_13 × 0.00274658));
y_7_2 = Logistic (0.622314+ (y_6_1 × − 0.276245) + (y_6_2 × 0.591492) + (y_6_3 × 0.660706) + (y_6_4 × − 0.858276) + (y_6_5 × 0.514343) + (y_6_6 × 0.303711) + (y_6_7 × − 0.849243) + (y_6_8 × − 0.845398) + (y_6_9 × 0.936951) + (y_6_10 × 0.921997) + (y_6_11 × − 0.171021) + (y_6_12 × 0.946411) + (y_6_13 × − 0.195618));
y_7_3 = Logistic (− 0.990784+ (y_6_1 × 0.422852) + (y_6_2 × 0.52063) + (y_6_3 × 0.776917) + (y_6_4 × 0.131592) + (y_6_5 × − 0.6203) + (y_6_6 × − 0.348877) + (y_6_7 × − 0.0666504) + (y_6_8 × − 0.633667) + (y_6_9 × 0.520508) + (y_6_10 × 0.822449) + (y_6_11 × − 0.748657) + (y_6_12 × − 0.923096) + (y_6_13 × − 0.085144));
y_7_4 = Logistic (− 0.746216+ (y_6_1 × 0.502808) + (y_6_2 × 0.295776) + (y_6_3 × 0.659424) + (y_6_4 × 0.101807) + (y_6_5 × − 0.107666) + (y_6_6 × 0.0143433) + (y_6_7 × 0.627197) + (y_6_8 × 0.472168) + (y_6_9 × − 0.97345) + (y_6_10 × 0.676453) + (y_6_11 × 0.0444946) + (y_6_12 × − 0.78302) + (y_6_13 × 0.181152));
y_7_5 = Logistic (− 0.735901+ (y_6_1 × − 0.0908813) + (y_6_2 × − 0.332642) + (y_6_3 × 0.154785) + (y_6_4 × 0.639587) + (y_6_5 × 0.644104) + (y_6_6 × − 0.645752) + (y_6_7 × 0.750549) + (y_6_8 × − 0.113159) + (y_6_9 × − 0.213318) + (y_6_10 × − 0.691833) + (y_6_11 × 0.638306) + (y_6_12 × − 0.708557) + (y_6_13 × − 0.579834));
y_7_6 = Logistic (− 0.878784+ (y_6_1 × − 0.286194) + (y_6_2 × 0.960266) + (y_6_3 × − 0.662292) + (y_6_4 × − 0.642151) + (y_6_5 × 0.331482) + (y_6_6 × − 0.165039) + (y_6_7 × 0.472168) + (y_6_8 × 0.855103) + (y_6_9 × 0.610657) + (y_6_10 × 0.266357) + (y_6_11 × 0.624878) + (y_6_12 × 0.294434) + (y_6_13 × − 0.11438));
y_7_7 = Logistic (0.525452+ (y_6_1 × − 0.192444) + (y_6_2 × − 0.530273) + (y_6_3 × 0.65509) + (y_6_4 × 0.215271) + (y_6_5 × 0.290344) + (y_6_6 × 0.485168) + (y_6_7 × − 0.418884) + (y_6_8 × − 0.569397) + (y_6_9 × 0.933716) + (y_6_10 × 0.878113) + (y_6_11 × − 0.141296) + (y_6_12 × 0.951172) + (y_6_13 × − 0.855774));
y_7_8 = Logistic (0.467102+ (y_6_1 × 0.434021) + (y_6_2 × − 0.883118) + (y_6_3 × − 0.0662842) + (y_6_4 × 0.319641) + (y_6_5 × − 0.645081) + (y_6_6 × 0.563171) + (y_6_7 × 0.315491) + (y_6_8 × 0.969604) + (y_6_9 × 0.148376) + (y_6_10 × − 0.433411) + (y_6_11 × 0.314453) + (y_6_12 × 0.464539) + (y_6_13 × − 0.370544));
y_7_9 = Logistic (− 0.06427+ (y_6_1 × − 0.0986938) + (y_6_2 × 0.949036) + (y_6_3 × − 0.273315) + (y_6_4 × 0.0991211) + (y_6_5 × 0.462524) + (y_6_6 × − 0.859924) + (y_6_7 × 0.174377) + (y_6_8 × − 0.239868) + (y_6_9 × 0.267761) + (y_6_10 × − 0.0431519) + (y_6_11 × − 0.766724) + (y_6_12 × − 0.565674) + (y_6_13 × 0.555847));
y_7_10 = Logistic (− 0.621765+ (y_6_1 × − 0.429138) + (y_6_2 × − 0.273926) + (y_6_3 × − 0.357239) + (y_6_4 × − 0.204285) + (y_6_5 × − 0.521606) + (y_6_6 × − 0.546021) + (y_6_7 × − 0.888123) + (y_6_8 × 0.418396) + (y_6_9 × − 0.444458) + (y_6_10 × 0.281677) + (y_6_11 × 0.767944) + (y_6_12 × − 0.916443) + (y_6_13 × 0.50769));
y_7_11 = Logistic (− 0.682861+ (y_6_1 × − 0.960876) + (y_6_2 × 0.282166) + (y_6_3 × − 0.705811) + (y_6_4 × − 0.723816) + (y_6_5 × − 0.447876) + (y_6_6 × − 0.770874) + (y_6_7 × 0.171204) + (y_6_8 × − 0.0359497) + (y_6_9 × 0.114014) + (y_6_10 × − 0.0789185) + (y_6_11 × 0.200745) + (y_6_12 × − 0.155823) + (y_6_13 × − 0.28833));
y_7_12 = Logistic (0.266846+ (y_6_1 × − 0.0195923) + (y_6_2 × − 0.233215) + (y_6_3 × − 0.988037) + (y_6_4 × − 0.682251) + (y_6_5 × − 0.28717) + (y_6_6 × − 0.718994) + (y_6_7 × − 0.755066) + (y_6_8 × − 0.438354) + (y_6_9 × 0.344177) + (y_6_10 × 0.723267) + (y_6_11 × 0.631714) + (y_6_12 × − 0.907593) + (y_6_13 × 0.182556));
y_7_13 = Logistic (− 0.578003+ (y_6_1 × − 0.595459) + (y_6_2 × − 0.466492) + (y_6_3 × 0.944702) + (y_6_4 × 0.769165) + (y_6_5 × − 0.235046) + (y_6_6 × 0.439453) + (y_6_7 × − 0.777832) + (y_6_8 × 0.0794678) + (y_6_9 × 0.0194702) + (y_6_10 × − 0.720215) + (y_6_11 × − 0.591248) + (y_6_12 × 0.838074) + (y_6_13 × − 0.53595));
y_8_1 = Logistic (− 0.124207+ (y_7_1 × 0.0327759) + (y_7_2 × 0.705505) + (y_7_3 × − 0.0281372) + (y_7_4 × 0.919434) + (y_7_5 × − 0.965393) + (y_7_6 × − 0.779114) + (y_7_7 × − 0.148743) + (y_7_8 × − 0.960022) + (y_7_9 × − 0.708252) + (y_7_10 × 0.909912) + (y_7_11 × 0.453613) + (y_7_12 × − 0.927795) + (y_7_13 × − 0.865662));
y_8_2 = Logistic (0.99707+ (y_7_1 × − 0.806946) + (y_7_2 × − 0.715881) + (y_7_3 × 0.200745) + (y_7_4 × 0.90802) + (y_7_5 × 0.335632) + (y_7_6 × 0.641663) + (y_7_7 × − 0.755249) + (y_7_8 × 0.0078125) + (y_7_9 × − 0.405579) + (y_7_10 × − 0.571289) + (y_7_11 × 0.478394) + (y_7_12 × − 0.332947) + (y_7_13 × 0.102783));
y_8_3 = Logistic (− 0.725159+ (y_7_1 × 0.746216) + (y_7_2 × 0.0958862) + (y_7_3 × − 0.188293) + (y_7_4 × − 0.443604) + (y_7_5 × 0.0861816) + (y_7_6 × 0.883789) + (y_7_7 × − 0.705872) + (y_7_8 × 0.762329) + (y_7_9 × − 0.0653687) + (y_7_10 × − 0.337585) + (y_7_11 × − 0.181824) + (y_7_12 × − 0.886047) + (y_7_13 × − 0.403137));
y_8_4 = Logistic (0.558533+ (y_7_1 × − 0.940186) + (y_7_2 × 0.868103) + (y_7_3 × − 0.946106) + (y_7_4 × 0.0581665) + (y_7_5 × 0.179749) + (y_7_6 × − 0.0460205) + (y_7_7 × 0.588379) + (y_7_8 × 0.692017) + (y_7_9 × − 0.27301) + (y_7_10 × − 0.51416) + (y_7_11 × − 0.0861816) + (y_7_12 × − 0.371582) + (y_7_13 × 0.152039));
y_8_5 = Logistic (0.230347+ (y_7_1 × 0.589478) + (y_7_2 × − 0.779724) + (y_7_3 × 0.595215) + (y_7_4 × 0.378174) + (y_7_5 × − 0.824036) + (y_7_6 × − 0.267456) + (y_7_7 × 0.0440674) + (y_7_8 × − 0.327454) + (y_7_9 × 0.577637) + (y_7_10 × − 0.947815) + (y_7_11 × − 0.625732) + (y_7_12 × 0.224548) + (y_7_13 × 0.0630493));
y_8_6 = Logistic (0.0239868+ (y_7_1 × 0.862854) + (y_7_2 × 0.0671387) + (y_7_3 × 0.993896) + (y_7_4 × − 0.456787) + (y_7_5 × 0.0614624) + (y_7_6 × 0.542297) + (y_7_7 × 0.390442) + (y_7_8 × − 0.218201) + (y_7_9 × − 0.622498) + (y_7_10 × 0.549866) + (y_7_11 × 0.348633) + (y_7_12 × 0.421753) + (y_7_13 × 0.543152));
y_8_7 = Logistic (− 0.917786+ (y_7_1 × − 0.090332) + (y_7_2 × − 0.640991) + (y_7_3 × − 0.859192) + (y_7_4 × 0.0769043) + (y_7_5 × − 0.588867) + (y_7_6 × − 0.980835) + (y_7_7 × 0.248596) + (y_7_8 × 0.742615) + (y_7_9 × 0.685425) + (y_7_10 × − 0.332886) + (y_7_11 × 0.0369873) + (y_7_12 × 0.747131) + (y_7_13 × 0.863037));
y_8_8 = Logistic (0.991577+ (y_7_1 × − 0.341614) + (y_7_2 × − 0.802002) + (y_7_3 × − 0.261414) + (y_7_4 × 0.828796) + (y_7_5 × − 0.148926) + (y_7_6 × − 0.380615) + (y_7_7 × − 0.332214) + (y_7_8 × 0.88739) + (y_7_9 × − 0.140259) + (y_7_10 × − 0.127686) + (y_7_11 × − 0.424255) + (y_7_12 × 0.65094) + (y_7_13 × − 0.358276));
y_8_9 = Logistic (− 0.192627+ (y_7_1 × 0.703186) + (y_7_2 × 0.369385) + (y_7_3 × 0.962341) + (y_7_4 × 0.977722) + (y_7_5 × 0.840881) + (y_7_6 × 0.647766) + (y_7_7 × 0.666138) + (y_7_8 × 0.40979) + (y_7_9 × 0.434021) + (y_7_10 × − 0.358154) + (y_7_11 × 0.0390625) + (y_7_12 × 0.198242) + (y_7_13 × 0.428955));
y_8_10 = Logistic (− 0.659485+ (y_7_1 × 0.329956) + (y_7_2 × − 0.306091) + (y_7_3 × 0.861267) + (y_7_4 × − 0.798096) + (y_7_5 × 0.791687) + (y_7_6 × 0.768127) + (y_7_7 × − 0.141418) + (y_7_8 × 0.757324) + (y_7_9 × 0.862122) + (y_7_10 × − 0.789917) + (y_7_11 × − 0.0174561) + (y_7_12 × − 0.194702) + (y_7_13 × 0.150146));
y_8_11 = Logistic (0.962769+ (y_7_1 × − 0.0240479) + (y_7_2 × 0.0181274) + (y_7_3 × 0.0306396) + (y_7_4 × 0.481445) + (y_7_5 × − 0.0514526) + (y_7_6 × − 0.764099) + (y_7_7 × 0.865112) + (y_7_8 × 0.830933) + (y_7_9 × − 0.608154) + (y_7_10 × 0.278503) + (y_7_11 × − 0.922974) + (y_7_12 × − 0.194946) + (y_7_13 × 0.75293));
y_8_12 = Logistic (− 0.0584106+ (y_7_1 × 0.00488281) + (y_7_2 × 0.488892) + (y_7_3 × − 0.261963) + (y_7_4 × 0.624084) + (y_7_5 × 0.85376) + (y_7_6 × 0.137024) + (y_7_7 × − 0.536743) + (y_7_8 × − 0.322144) + (y_7_9 × 0.289246) + (y_7_10 × 0.184814) + (y_7_11 × − 0.176392) + (y_7_12 × 0.386719) + (y_7_13 × 0.890869));
y_8_13 = Logistic (0.935974+ (y_7_1 × − 0.0975342) + (y_7_2 × 0.981995) + (y_7_3 × − 0.659119) + (y_7_4 × 0.650696) + (y_7_5 × − 0.974304) + (y_7_6 × − 0.404419) + (y_7_7 × − 0.0240479) + (y_7_8 × 0.039978) + (y_7_9 × 0.805664) + (y_7_10 × 0.91571) + (y_7_11 × 0.523682) + (y_7_12 × − 0.592041) + (y_7_13 × 0.0631104));
y_9_1 = Logistic (0.0827637+ (y_8_1 × 0.756836) + (y_8_2 × 0.0921631) + (y_8_3 × − 0.937622) + (y_8_4 × 0.112976) + (y_8_5 × − 0.591187) + (y_8_6 × 0.185364) + (y_8_7 × 0.842957) + (y_8_8 × − 0.0303345) + (y_8_9 × 0.106384) + (y_8_10 × − 0.300171) + (y_8_11 × 0.247742) + (y_8_12 × − 0.397644) + (y_8_13 × 0.592957));
y_9_2 = Logistic (− 0.386108+ (y_8_1 × − 0.691406) + (y_8_2 × 0.547607) + (y_8_3 × 0.328308) + (y_8_4 × 0.5271) + (y_8_5 × 0.2052) + (y_8_6 × 0.655029) + (y_8_7 × 0.930237) + (y_8_8 × − 0.000854492) + (y_8_9 × − 0.196838) + (y_8_10 × 0.276062) + (y_8_11 × − 0.548523) + (y_8_12 × 0.395874) + (y_8_13 × 0.392273));
y_9_3 = Logistic (− 0.134827+ (y_8_1 × 0.190552) + (y_8_2 × − 0.395813) + (y_8_3 × − 0.57489) + (y_8_4 × − 0.685181) + (y_8_5 × − 0.026001) + (y_8_6 × 0.854065) + (y_8_7 × − 0.886963) + (y_8_8 × 0.223694) + (y_8_9 × 0.473755) + (y_8_10 × 0.242249) + (y_8_11 × 0.586792) + (y_8_12 × − 0.0152588) + (y_8_13 × − 0.64917));
y_9_4 = Logistic (− 0.99762+ (y_8_1 × − 0.990723) + (y_8_2 × 0.454346) + (y_8_3 × 0.78656) + (y_8_4 × − 0.998657) + (y_8_5 × 0.0809937) + (y_8_6 × 0.594971) + (y_8_7 × 0.0197144) + (y_8_8 × − 0.831055) + (y_8_9 × − 0.518555) + (y_8_10 × 0.172241) + (y_8_11 × − 0.512451) + (y_8_12 × − 0.438721) + (y_8_13 × − 0.266846));
y_9_5 = Logistic (− 0.357178+ (y_8_1 × − 0.318176) + (y_8_2 × 0.51239) + (y_8_3 × 0.782837) + (y_8_4 × 0.308716) + (y_8_5 × − 0.152466) + (y_8_6 × 0.498047) + (y_8_7 × 0.89679) + (y_8_8 × − 0.804565) + (y_8_9 × − 0.822815) + (y_8_10 × − 0.487976) + (y_8_11 × − 0.0718994) + (y_8_12 × 0.635925) + (y_8_13 × 0.991089));
y_9_6 = Logistic (− 0.611511+ (y_8_1 × 0.44989) + (y_8_2 × − 0.379395) + (y_8_3 × − 0.893433) + (y_8_4 × − 0.264343) + (y_8_5 × − 0.655762) + (y_8_6 × 0.760498) + (y_8_7 × 0.597412) + (y_8_8 × − 0.604919) + (y_8_9 × − 0.61969) + (y_8_10 × − 0.621277) + (y_8_11 × − 0.745239) + (y_8_12 × − 0.187378) + (y_8_13 × − 0.81842));
y_9_7 = Logistic (0.680908+ (y_8_1 × 0.144897) + (y_8_2 × 0.449829) + (y_8_3 × − 0.265076) + (y_8_4 × 0.927307) + (y_8_5 × 0.264771) + (y_8_6 × 0.335693) + (y_8_7 × 0.391235) + (y_8_8 × 0.864014) + (y_8_9 × 0.19458) + (y_8_10 × 0.954956) + (y_8_11 × 0.420532) + (y_8_12 × − 0.224915) + (y_8_13 × 0.483948));
y_9_8 = Logistic (− 0.375244+ (y_8_1 × 0.960571) + (y_8_2 × − 0.0413818) + (y_8_3 × − 0.0311279) + (y_8_4 × − 0.939758) + (y_8_5 × − 0.987427) + (y_8_6 × 0.698059) + (y_8_7 × 0.349426) + (y_8_8 × 0.26709) + (y_8_9 × − 0.0866699) + (y_8_10 × − 0.267212) + (y_8_11 × 0.13147) + (y_8_12 × − 0.0311279) + (y_8_13 × 0.543091));
y_9_9 = Logistic (0.102234+ (y_8_1 × 0.00134277) + (y_8_2 × 0.762634) + (y_8_3 × 0.034668) + (y_8_4 × − 0.34082) + (y_8_5 × − 0.861023) + (y_8_6 × 0.615173) + (y_8_7 × 0.512939) + (y_8_8 × 0.620605) + (y_8_9 × − 0.446411) + (y_8_10 × 0.00933838) + (y_8_11 × 0.810913) + (y_8_12 × − 0.898254) + (y_8_13 × − 0.463867));
y_9_10 = Logistic (0.425354+ (y_8_1 × − 0.868103) + (y_8_2 × 0.189575) + (y_8_3 × 0.85553) + (y_8_4 × 0.218811) + (y_8_5 × 0.411377) + (y_8_6 × 0.888) + (y_8_7 × − 0.40863) + (y_8_8 × 0.193726) + (y_8_9 × − 0.0875244) + (y_8_10 × 0.189331) + (y_8_11 × 0.0274658) + (y_8_12 × 0.603577) + (y_8_13 × − 0.686646));
y_9_11 = Logistic (0.141663+ (y_8_1 × 0.739868) + (y_8_2 × 0.182617) + (y_8_3 × 0.51825) + (y_8_4 × − 0.973389) + (y_8_5 × − 0.564453) + (y_8_6 × − 0.0319824) + (y_8_7 × − 0.591675) + (y_8_8 × 0.0278931) + (y_8_9 × 0.186462) + (y_8_10 × 0.142395) + (y_8_11 × 0.529541) + (y_8_12 × 0.953613) + (y_8_13 × 0.873291));
y_9_12 = Logistic (0.0795288+ (y_8_1 × 0.693604) + (y_8_2 × 0.0290527) + (y_8_3 × 0.0310059) + (y_8_4 × − 0.192383) + (y_8_5 × 0.707458) + (y_8_6 × − 0.412354) + (y_8_7 × 0.402527) + (y_8_8 × 0.00842285) + (y_8_9 × 0.788269) + (y_8_10 × − 0.591064) + (y_8_11 × 0.407288) + (y_8_12 × 0.607483) + (y_8_13 × 0.0922241));
y_9_13 = Logistic (0.574768+ (y_8_1 × − 0.630737) + (y_8_2 × − 0.617676) + (y_8_3 × − 0.303406) + (y_8_4 × − 0.96344) + (y_8_5 × − 0.860168) + (y_8_6 × 0.545288) + (y_8_7 × − 0.0718994) + (y_8_8 × − 0.253052) + (y_8_9 × − 0.905701) + (y_8_10 × − 0.4776) + (y_8_11 × − 0.817566) + (y_8_12 × − 0.693848) + (y_8_13 × 0.813843));
y_10_1 = Logistic (0.277649+ (y_9_1 × 0.454407) + (y_9_2 × 0.843506) + (y_9_3 × 0.21991) + (y_9_4 × − 0.424072) + (y_9_5 × 0.70343) + (y_9_6 × − 0.219849) + (y_9_7 × 0.0346069) + (y_9_8 × − 0.769531) + (y_9_9 × − 0.47406) + (y_9_10 × 0.708252) + (y_9_11 × 0.801453) + (y_9_12 × − 0.93866) + (y_9_13 × − 0.716553));
y_10_2 = Logistic (− 0.323425+ (y_9_1 × 0.751343) + (y_9_2 × − 0.473999) + (y_9_3 × − 0.0760498) + (y_9_4 × 0.793945) + (y_9_5 × 0.304138) + (y_9_6 × − 0.521912) + (y_9_7 × − 0.353455) + (y_9_8 × 0.659241) + (y_9_9 × 0.300598) + (y_9_10 × 0.638123) + (y_9_11 × 0.94104) + (y_9_12 × − 0.21637) + (y_9_13 × − 0.0927124));
y_10_3 = Logistic (− 0.657227+ (y_9_1 × 0.866577) + (y_9_2 × − 0.854675) + (y_9_3 × 0.396912) + (y_9_4 × 0.705566) + (y_9_5 × − 0.39209) + (y_9_6 × 0.360046) + (y_9_7 × − 0.10675) + (y_9_8 × 0.424011) + (y_9_9 × 0.943176) + (y_9_10 × − 0.457275) + (y_9_11 × 0.799377) + (y_9_12 × 0.284424) + (y_9_13 × − 0.198425));
y_10_4 = Logistic (0.750183+ (y_9_1 × − 0.87854) + (y_9_2 × − 0.816284) + (y_9_3 × − 0.976379) + (y_9_4 × 0.145508) + (y_9_5 × − 0.309814) + (y_9_6 × 0.820129) + (y_9_7 × − 0.34668) + (y_9_8 × 0.940796) + (y_9_9 × − 0.651062) + (y_9_10 × − 0.582825) + (y_9_11 × 0.0210571) + (y_9_12 × 0.723877) + (y_9_13 × − 0.749084));
y_10_5 = Logistic (0.324707+ (y_9_1 × 0.227295) + (y_9_2 × − 0.765747) + (y_9_3 × 0.579834) + (y_9_4 × − 0.0808716) + (y_9_5 × − 0.653687) + (y_9_6 × − 0.990051) + (y_9_7 × 0.342834) + (y_9_8 × − 0.104797) + (y_9_9 × 0.0177612) + (y_9_10 × 0.0130615) + (y_9_11 × − 0.168396) + (y_9_12 × − 0.717712) + (y_9_13 × 0.253235));
y_10_6 = Logistic (0.166931+ (y_9_1 × 0.245178) + (y_9_2 × − 0.0671997) + (y_9_3 × − 0.254272) + (y_9_4 × − 0.91864) + (y_9_5 × − 0.453003) + (y_9_6 × − 0.65564) + (y_9_7 × − 0.46759) + (y_9_8 × − 0.261169) + (y_9_9 × − 0.723999) + (y_9_10 × − 0.208313) + (y_9_11 × − 0.927124) + (y_9_12 × − 0.885315) + (y_9_13 × − 0.777161));
y_10_7 = Logistic (0.0745239+ (y_9_1 × − 0.471436) + (y_9_2 × − 0.272461) + (y_9_3 × − 0.238464) + (y_9_4 × 0.828491) + (y_9_5 × − 0.0635986) + (y_9_6 × − 0.759888) + (y_9_7 × − 0.214661) + (y_9_8 × 0.764343) + (y_9_9 × − 0.96698) + (y_9_10 × 0.460327) + (y_9_11 × 0.926575) + (y_9_12 × − 0.0614624) + (y_9_13 × 0.704407));
y_10_8 = Logistic (− 0.346375+ (y_9_1 × 0.899109) + (y_9_2 × 0.145874) + (y_9_3 × − 0.00970459) + (y_9_4 × − 0.528381) + (y_9_5 × − 0.322021) + (y_9_6 × − 0.975037) + (y_9_7 × 0.3349) + (y_9_8 × − 0.394775) + (y_9_9 × − 0.564697) + (y_9_10 × 0.297913) + (y_9_11 × − 0.177734) + (y_9_12 × 0.399963) + (y_9_13 × − 0.247192));
y_10_9 = Logistic (− 0.802917+ (y_9_1 × − 0.17749) + (y_9_2 × 0.0714111) + (y_9_3 × 0.422119) + (y_9_4 × − 0.336975) + (y_9_5 × − 0.693604) + (y_9_6 × 0.54248) + (y_9_7 × 0.541809) + (y_9_8 × − 0.19751) + (y_9_9 × 0.098999) + (y_9_10 × 0.410461) + (y_9_11 × 0.563477) + (y_9_12 × − 0.229126) + (y_9_13 × − 0.636841));
y_10_10 = Logistic (0.164734+ (y_9_1 × − 0.698486) + (y_9_2 × − 0.734192) + (y_9_3 × − 0.739563) + (y_9_4 × 0.937195) + (y_9_5 × − 0.0446167) + (y_9_6 × 0.664734) + (y_9_7 × 0.517029) + (y_9_8 × 0.201538) + (y_9_9 × 0.876099) + (y_9_10 × 0.311768) + (y_9_11 × 0.695984) + (y_9_12 × − 0.73822) + (y_9_13 × 0.0333252));
y_10_11 = Logistic (0.146667+ (y_9_1 × 0.439514) + (y_9_2 × − 0.325439) + (y_9_3 × − 0.239868) + (y_9_4 × − 0.322876) + (y_9_5 × − 0.232422) + (y_9_6 × − 0.115295) + (y_9_7 × − 0.371277) + (y_9_8 × − 0.671692) + (y_9_9 × 0.781311) + (y_9_10 × − 0.273193) + (y_9_11 × − 0.903198) + (y_9_12 × − 0.992737) + (y_9_13 × 0.295471));
y_10_12 = Logistic (− 0.490173+ (y_9_1 × − 0.871094) + (y_9_2 × 0.264038) + (y_9_3 × − 0.236389) + (y_9_4 × 0.115356) + (y_9_5 × 0.8125) + (y_9_6 × − 0.44696) + (y_9_7 × − 0.431091) + (y_9_8 × − 0.391296) + (y_9_9 × − 0.17218) + (y_9_10 × − 0.646912) + (y_9_11 × − 0.364929) + (y_9_12 × 0.999695) + (y_9_13 × 0.921326));
y_10_13 = Logistic (0.602112+ (y_9_1 × 0.167786) + (y_9_2 × − 0.778259) + (y_9_3 × − 0.801758) + (y_9_4 × 0.297729) + (y_9_5 × − 0.420715) + (y_9_6 × − 0.00653076) + (y_9_7 × 0.977112) + (y_9_8 × − 0.661011) + (y_9_9 × − 0.658569) + (y_9_10 × − 0.0090332) + (y_9_11 × − 0.690918) + (y_9_12 × 0.842712) + (y_9_13 × 0.913757));
y_11_1 = Logistic (− 0.703979+ (y_10_1 × − 0.545166) + (y_10_2 × 0.483154) + (y_10_3 × − 0.288086) + (y_10_4 × 0.864868) + (y_10_5 × 0.547607) + (y_10_6 × 0.0967407) + (y_10_7 × 0.589905) + (y_10_8 × − 0.109009) + (y_10_9 × 0.0396729) + (y_10_10 × − 0.742798) + (y_10_11 × 0.155823) + (y_10_12 × 0.180603) + (y_10_13 × − 0.177917));
y_11_2 = Logistic (0.441772+ (y_10_1 × − 0.883484) + (y_10_2 × − 0.522095) + (y_10_3 × − 0.0443726) + (y_10_4 × − 0.0512695) + (y_10_5 × 0.353821) + (y_10_6 × 0.996399) + (y_10_7 × − 0.53717) + (y_10_8 × − 0.022583) + (y_10_9 × 0.402039) + (y_10_10 × − 0.906921) + (y_10_11 × 0.514832) + (y_10_12 × 0.59613) + (y_10_13 × − 0.180603));
y_11_3 = Logistic (− 0.411255+ (y_10_1 × − 0.772705) + (y_10_2 × 0.332336) + (y_10_3 × 0.346924) + (y_10_4 × − 0.632385) + (y_10_5 × 0.950684) + (y_10_6 × − 0.250671) + (y_10_7 × − 0.740234) + (y_10_8 × 0.359863) + (y_10_9 × − 0.88623) + (y_10_10 × 0.844482) + (y_10_11 × 0.209412) + (y_10_12 × − 0.159241) + (y_10_13 × 0.575928));
y_11_4 = Logistic (− 0.621277+ (y_10_1 × 0.0600586) + (y_10_2 × − 0.192688) + (y_10_3 × − 0.839294) + (y_10_4 × 0.732056) + (y_10_5 × − 0.127502) + (y_10_6 × − 0.35907) + (y_10_7 × − 0.492371) + (y_10_8 × − 0.831848) + (y_10_9 × − 0.924377) + (y_10_10 × − 0.326599) + (y_10_11 × 0.957581) + (y_10_12 × 0.758118) + (y_10_13 × 0.739868));
y_11_5 = Logistic (0.365906+ (y_10_1 × 0.616028) + (y_10_2 × 0.608276) + (y_10_3 × − 0.485229) + (y_10_4 × − 0.0899048) + (y_10_5 × − 0.914368) + (y_10_6 × 0.356384) + (y_10_7 × − 0.493103) + (y_10_8 × 0.103455) + (y_10_9 × 0.359619) + (y_10_10 × − 0.117859) + (y_10_11 × 0.155457) + (y_10_12 × 0.671265) + (y_10_13 × 0.541504));
y_11_6 = Logistic (− 0.816406+ (y_10_1 × − 0.596252) + (y_10_2 × 0.221252) + (y_10_3 × 0.0861206) + (y_10_4 × 0.948486) + (y_10_5 × − 0.236816) + (y_10_6 × − 0.203796) + (y_10_7 × − 0.782166) + (y_10_8 × 0.534851) + (y_10_9 × 0.407349) + (y_10_10 × 0.611206) + (y_10_11 × − 0.340454) + (y_10_12 × − 0.837402) + (y_10_13 × 0.825317));
y_11_7 = Logistic (− 0.158997+ (y_10_1 × − 0.0269165) + (y_10_2 × − 0.832275) + (y_10_3 × − 0.467651) + (y_10_4 × 0.00604248) + (y_10_5 × 0.6427) + (y_10_6 × − 0.722778) + (y_10_7 × 0.586975) + (y_10_8 × − 0.522461) + (y_10_9 × − 0.0331421) + (y_10_10 × − 0.84436) + (y_10_11 × 0.200195) + (y_10_12 × 0.490479) + (y_10_13 × 0.576965));
y_11_8 = Logistic (0.192627+ (y_10_1 × 0.757141) + (y_10_2 × − 0.0368652) + (y_10_3 × 0.909424) + (y_10_4 × − 0.0335083) + (y_10_5 × − 0.659058) + (y_10_6 × − 0.119385) + (y_10_7 × 0.373047) + (y_10_8 × − 0.740479) + (y_10_9 × 0.414429) + (y_10_10 × − 0.209351) + (y_10_11 × − 0.713562) + (y_10_12 × 0.483582) + (y_10_13 × − 0.834534));
y_11_9 = Logistic (0.200317+ (y_10_1 × − 0.68158) + (y_10_2 × 0.208374) + (y_10_3 × 0.658936) + (y_10_4 × − 0.55542) + (y_10_5 × − 0.821411) + (y_10_6 × − 0.804871) + (y_10_7 × 0.0163574) + (y_10_8 × 0.361328) + (y_10_9 × − 0.362549) + (y_10_10 × − 0.336182) + (y_10_11 × 0.656494) + (y_10_12 × − 0.444153) + (y_10_13 × 0.947449));
y_11_10 = Logistic (− 0.112793+ (y_10_1 × 0.33844) + (y_10_2 × 0.96344) + (y_10_3 × − 0.791138) + (y_10_4 × − 0.432556) + (y_10_5 × − 0.160522) + (y_10_6 × 0.0845337) + (y_10_7 × − 0.147644) + (y_10_8 × 0.980164) + (y_10_9 × − 0.518799) + (y_10_10 × − 0.211548) + (y_10_11 × 0.0440063) + (y_10_12 × 0.920227) + (y_10_13 × 0.838562));
y_11_11 = Logistic (0.00408936+ (y_10_1 × 0.0160522) + (y_10_2 × 0.966736) + (y_10_3 × − 0.17572) + (y_10_4 × − 0.354675) + (y_10_5 × − 0.566406) + (y_10_6 × 0.0167847) + (y_10_7 × 0.316711) + (y_10_8 × 0.58783) + (y_10_9 × − 0.696411) + (y_10_10 × 0.442932) + (y_10_11 × 0.542969) + (y_10_12 × 0.554993) + (y_10_13 × − 0.0161133));
scaled_Belgium = (− 0.666443+ (y_11_1 × 0.750977) + (y_11_2 × 0.38208) + (y_11_3 × 0.535828) + (y_11_4 × − 0.817627) + (y_11_5 × 0.172241) + (y_11_6 × 0.655396) + (y_11_7 × − 0.875671) + (y_11_8 × 0.133423) + (y_11_9 × 0.345764) + (y_11_10 × − 0.472107) + (y_11_11 × 0.909607) + (y_11_12 × − 0.176758));
scaled_Finland = (0.484009+ (y_11_1 × − 0.622131) + (y_11_2 × 0.41217) + (y_11_3 × − 0.540466) + (y_11_4 × − 0.868958) + (y_11_5 × 0.881897) + (y_11_6 × 0.71521) + (y_11_7 × 0.812256) + (y_11_8 × 0.166382) + (y_11_9 × 0.858459) + (y_11_10 × 0.447815) + (y_11_11 × − 0.102905) + (y_11_12 × 0.255615));
scaled_France = (− 0.135132+ (y_11_1 × − 0.0143433) + (y_11_2 × 0.604004) + (y_11_3 × − 0.352966) + (y_11_4 × − 0.14563) + (y_11_5 × 0.132141) + (y_11_6 × 0.872009) + (y_11_7 × 0.115479) + (y_11_8 × 0.447998) + (y_11_9 × 0.904907) + (y_11_10 × − 0.759888) + (y_11_11 × − 0.0911255) + (y_11_12 × − 0.496643));
scaled_Germany = (0.740173+ (y_11_1 × − 0.954651) + (y_11_2 × − 0.940674) + (y_11_3 × − 0.751343) + (y_11_4 × 0.533081) + (y_11_5 × 0.485168) + (y_11_6 × 0.125793) + (y_11_7 × 0.974121) + (y_11_8 × − 0.663635) + (y_11_9 × − 0.125427) + (y_11_10 × − 0.655212) + (y_11_11 × 0.799805) + (y_11_12 × 0.4776));
scaled_Greece = (− 0.483459+ (y_11_1 × − 0.603455) + (y_11_2 × − 0.460144) + (y_11_3 × − 0.861023) + (y_11_4 × − 0.934143) + (y_11_5 × − 0.322327) + (y_11_6 × − 0.0314331) + (y_11_7 × − 0.194214) + (y_11_8 × − 0.102051) + (y_11_9 × 0.610107) + (y_11_10 × − 0.891968) + (y_11_11 × − 0.0908813) + (y_11_12 × − 0.973389));
scaled_Ireland = (0.612488+ (y_11_1 × 0.895569) + (y_11_2 × − 0.728027) + (y_11_3 × 0.250671) + (y_11_4 × − 0.182007) + (y_11_5 × 0.124634) + (y_11_6 × − 0.376709) + (y_11_7 × 0.538452) + (y_11_8 × 0.558594) + (y_11_9 × 0.203857) + (y_11_10 × − 0.857666) + (y_11_11 × 0.730347) + (y_11_12 × − 0.32312));
scaled_Italy = (− 0.845581+ (y_11_1 × − 0.0570679) + (y_11_2 × 0.11615) + (y_11_3 × 0.818237) + (y_11_4 × − 0.360413) + (y_11_5 × − 0.385681) + (y_11_6 × − 0.291565) + (y_11_7 × 0.366272) + (y_11_8 × − 0.413086) + (y_11_9 × − 0.701477) + (y_11_10 × 0.175049) + (y_11_11 × 0.116028) + (y_11_12 × − 0.23175));
scaled_Luxembourg = (0.725891+ (y_11_1 × − 0.173523) + (y_11_2 × − 0.714172) + (y_11_3 × 0.969543) + (y_11_4 × − 0.158752) + (y_11_5 × 0.132324) + (y_11_6 × − 0.483521) + (y_11_7 × 0.664978) + (y_11_8 × 0.0966797) + (y_11_9 × − 0.170959) + (y_11_10 × − 0.427856) + (y_11_11 × − 0.544922) + (y_11_12 × − 0.328308));
scaled_Netherland = (− 0.640198+ (y_11_1 × 0.818909) + (y_11_2 × 0.805542) + (y_11_3 × − 0.897156) + (y_11_4 × 0.813477) + (y_11_5 × 0.75177) + (y_11_6 × 0.0924072) + (y_11_7 × 0.505554) + (y_11_8 × − 0.743286) + (y_11_9 × 0.143433) + (y_11_10 × − 0.273987) + (y_11_11 × 0.260803) + (y_11_12 × − 0.223633));
scaled_Portugal = (− 0.408325+ (y_11_1 × − 0.0332642) + (y_11_2 × − 0.105652) + (y_11_3 × − 0.514404) + (y_11_4 × 0.979065) + (y_11_5 × − 0.472168) + (y_11_6 × 0.300964) + (y_11_7 × − 0.79718) + (y_11_8 × 0.395081) + (y_11_9 × − 0.559937) + (y_11_10 × 0.493774) + (y_11_11 × − 0.394226) + (y_11_12 × − 0.0663452));
scaled_Spain = (0.95929+ (y_11_1 × − 0.492188) + (y_11_2 × − 0.537354) + (y_11_3 × − 0.485352) + (y_11_4 × 0.76416) + (y_11_5 × 0.333374) + (y_11_6 × 0.0697021) + (y_11_7 × − 0.663391) + (y_11_8 × − 0.314575) + (y_11_9 × 0.458862) + (y_11_10 × 0.873291) + (y_11_11 × − 0.157349) + (y_11_12 × 0.0458374));
scaled_Latentfactor = (0.274963+ (y_11_1 × − 0.14447) + (y_11_2 × 0.909912) + (y_11_3 × 0.0727539) + (y_11_4 × − 0.723022) + (y_11_5 × − 0.426575) + (y_11_6 × − 0.00500488) + (y_11_7 × 0.683167) + (y_11_8 × 0.102661) + (y_11_9 × 0.363892) + (y_11_10 × 0.106079) + (y_11_11 × 0.0469971) + (y_11_12 × − 0.513428));
(Belgium,Finland,France,Germany,Greece,Ireland,Italy,Luxembourg,Netherland ,Portugal,Spain,Latentfactor,LnAustria,LnBelgium,LnFiland,LnFrance,LnGermany) = (0.5 × (scaled_Belgium + 1.0) × (45.04 − 42.37) + 42.37,0.5 × (scaled_Finland+1.0) × (43.988 − 40.788) + 40.788,0.5 × (scaled_France + 1.0) × (45.504 − 41.323) + 41.323,0.5 × (scaled_Germany + 1.0) × (36.937 − 33.86)+33.86,0.5 × (scaled_Greece + 1.0) × (36.78 − 29.917) + 29.917,0.5 × (scaled_Ireland + 1.0) × (30.82 − 23.594) + 23.594,0.5 × (scaled_Italy + 1.0) × (43.973 − 39.149) + 39.149,0.5 × (scaled_Luxembourg + 1.0) × (38.947 − 36.15) + 36.15,0.5 × (scaled_Netherland + 1.0) × (37.751 − 35.164) + 35.164,0.5 × (scaled_Portugal + 1.0) × (34.489 − 29.914) + 29.914,0.5 × (scaled_Spain + 1.0) × (36.53 − 29.956) + 29.956,0.5 × (scaled_Latentfactor + 1.0) × (6.49091 + 2.61157) − 2.61157,0.5 × (scaled_LnAustria + 1.0) × (3.77794 − 3.69804) + 3.69804,0.5 × (scaled_LnBelgium + 1.0) × (3.80755 − 3.71262) + 3.71262,0.5 × (scaled_LnFiland + 1.0) × (3.82472 − 3.70839) + 3.70839,0.5 × (scaled_LnFrance + 1.0) × (3.8178 − 3.71213) + 3.71213,0.5 × (scaled_LnGermany + 1.0) × (3.60921 − 3.52223) + 3.52223);
logistic(x){
return 1/(1+exp(− x))

## Abbreviations

AMECO: Annual Macro-Economic Database
ANNs: Artificial neural networks
BPNN: Back-propagation neural networks
DFM: Dynamic factor model
DG ECFIN: European Commission’s Directorate General for Economic and Financial Affairs
ECOFIN: Economic and Financial Affairs Council
EMU: European Monetary Union
EU: European Union
GDP: Gross domestic product
GGDC: Groningen Growth and Development Centre
ML: Machine learning
NN: Neural network
OCA: Optimal currency area
PDG: Projected descending gradient
PWT: Penn world table
VAR: Vector auto regression

## Footnotes

1 https://www.rug.nl/ggdc/productivity/pwt/.

2 https://ec.europa.eu/info/business-economy-euro/indicators-statistics/economic-databases/macro-economic-database-ameco/download-annual-data-set-macro-economic-database-ameco_en.

3 Result = *DR*_*n,k*_. In this case, *k*, a positive integer, can also be greater than or equal to *n*.

4 The NN process automatically eliminated Austria: the hidden values of this variable generated errors in gradient descents.
